# Heinz-resistant tomato cultivars exhibit a lignin-based resistance to field dodder (*Cuscuta campestris*) parasitism

**DOI:** 10.1093/plphys/kiac024

**Published:** 2022-01-31

**Authors:** Min-Yao Jhu, Moran Farhi, Li Wang, Richard N Philbrook, Michael S Belcher, Hokuto Nakayama, Kristina S Zumstein, Sarah D Rowland, Mily Ron, Patrick M Shih, Neelima R Sinha

**Affiliations:** Department of Plant Biology, University of California, Davis, CA 95616, USA; Crop Science Centre, Department of Plant Sciences, University of Cambridge, Cambridge, UK; Department of Plant Biology, University of California, Davis, CA 95616, USA; The Better Meat Co., 2939 Promenade St. West Sacramento, CA 95691, USA; Department of Plant Biology, University of California, Davis, CA 95616, USA; College of Life Sciences, Nanjing Normal University, Nanjing, Jiangsu, China; Department of Plant Biology, University of California, Davis, CA 95616, USA; Dark Heart Nursery, 630 Pena Dr, Davis, CA 95616, USA; Feedstocks Division, Joint BioEnergy Institute, Emeryville, CA 94608, USA; Department of Plant and Microbial Biology, University of California, Berkeley, CA, USA; Department of Plant Biology, University of California, Davis, CA 95616, USA; Graduate School of Science, Department of Biological Sciences, University of Tokyo, Hongo Bunkyo-ku, Tokyo, 113-0033, Japan; Department of Plant Biology, University of California, Davis, CA 95616, USA; Department of Plant Biology, University of California, Davis, CA 95616, USA; Department of Plant Biology, University of California, Davis, CA 95616, USA; Department of Plant Biology, University of California, Davis, CA 95616, USA; Feedstocks Division, Joint BioEnergy Institute, Emeryville, CA 94608, USA; Genome Center, University of California, Davis, CA 95616, USA; Environmental Genomics and Systems Biology Division, Lawrence Berkeley National Laboratory, Berkeley, CA 94720, USA; Department of Plant Biology, University of California, Davis, CA 95616, USA

## Abstract

*Cuscuta* species (dodders) are agriculturally destructive, parasitic angiosperms. These parasitic plants use haustoria as physiological bridges to extract nutrients and water from hosts. *Cuscuta campestris* has a broad host range and wide geographical distribution. While some wild tomato relatives are resistant, cultivated tomatoes are generally susceptible to *C. campestris* infestations. However, some specific Heinz tomato (*Solanum lycopersicum*) hybrid cultivars exhibit resistance to dodders in the field, but their defense mechanism was previously unknown. Here, we discovered that the stem cortex in these resistant lines responds with local lignification upon *C. campestris* attachment, preventing parasite entry into the host. *Lignin Induction Factor 1* (*LIF1*, an *AP2*-like transcription factor), *SlMYB55*, and *Cuscuta R-gene for Lignin-based Resistance 1*, a *CC-NBS-LRR* (*CuRLR1*) are identified as factors that confer host resistance by regulating lignification. *SlWRKY16* is upregulated upon *C. campestris* infestation and potentially negatively regulates *LIF1* function. Intriguingly, *CuRLR1* may play a role in signaling or function as an intracellular receptor for receiving *Cuscuta* signals or effectors, thereby regulating lignification-based resistance. In summary, these four regulators control the lignin-based resistance response in specific Heinz tomato cultivars, preventing *C. campestris* from parasitizing resistant tomatoes. This discovery provides a foundation for investigating multilayer resistance against *Cuscuta* species and has potential for application in other essential crops attacked by parasitic plants.

## Teaser

Four key regulators confer lignin accumulation in the tomato stem cortex to block host penetration upon field dodder (*Cuscuta campestris*) infection.

## Introduction

Parasitic plants directly attach to hosts using specialized haustorial organs known as haustoria. These connections function as physiological bridges to extract nutrients and water from the hosts, making most traditional herbicides and control methods, including management of soil fertility, hand weeding, and sanitation, either too cost-intensive, labor-intensive, or ineffective in regulating parasitic plant infestations. Therefore, parasitic angiosperms are among the most devastating pests, reducing the yields of agricultural crops each year by billions of dollars worldwide (Agrios, 2005; Yoder and Scholes, 2010). Members of the *Cuscuta* genus (family Convolvulaceae), also known as dodders, occur worldwide and *Cuscuta* infestations in tomato (*Solanum lycopersicum*) alone lead to 50%–72% yield reductions (Goldwasser et al., 2001). Despite serious agricultural problems caused by *Cuscuta*, our understanding of the interactions between *Cuscuta* and its hosts is relatively limited compared to our knowledge of pathogenic fungi, bacteria, and viruses. Only recently, the first receptor (*CUSCUTA RECEPTOR 1*, *CuRe1*, Solyc08g016270), an LRR receptor-like serine/threonine-protein kinase (RLP), from *Cuscuta* was identified in tomatoes (Hegenauer et al., 2016, 2020). *CuRe1* initiates pathogen-associated molecular pattern (PAMP)-triggered immunity to *Cuscuta reflexa* (Hegenauer et al., 2016, 2020). However, plants that lack *CuRe1* are still fully resistant to *C. reflexa*. This result indicates that other layers of defense, besides *CuRe1*, must also be involved in the responses to these parasites. Therefore, further investigating the potential multilayered resistance mechanisms will aid in developing parasitic plant-resistant crops.

Potential immune responses and defense responses to parasitic plants have been observed in several crop plants, including rice (*Oryza sativa*), tomato, cowpea (*Vigna unguiculata*), and sunflower (*Helianthus annuus*) (Mutuku et al., 2015; Hegenauer et al., 2016; Duriez et al., 2019; Su et al., 2020). Notably, most previous reports indicated that hypersensitive response is the major mechanism that contributes to plant immunity to parasitic plants (Lane et al., 1993; Hegenauer et al., 2016; Su et al., 2020). A few studies indicated that secondary cell-wall modification and formation in the resistant rice (*Oryza sativa*) cultivar “Nipponbare” also contribute to defense against root parasitic plants, like *Striga hermonthica* (Yoshida and Shirasu, 2009; Mutuku et al., 2019). Plants often modify their cell walls in response to pathogen infection and herbivore feeding (Moura et al., 2010). Among different modifications, lignification is considered a major mechanism for resistance in plants (Vance et al., 1980; Moura et al., 2010; Bellincampi et al., 2014; Malinovsky et al., 2014). Lignified cell walls have higher mechanical strength, are impermeable to water, and less accessible to cell wall-degrading enzymes (Bhuiyan et al., 2009; Barros et al., 2015). Several previous reports indicated that lignified endodermal cells were found in resistant host roots, like vetch (*Vicia* spp.) and faba bean (*Vicia faba*), in response to root parasitic plant attack (Pérez-de-Luque et al., 2005, 2007). However, how secondary cell wall modification and lignin are involved in the defense responses to stem parasitic plants still needs to be elucidated. Thus, we specifically investigated host cell wall composition and the lignin biosynthesis pathways aiming to discover the potential additional layers of resistance to *Cuscuta* spp.

*Cuscuta campestris* attacks a wide range of crop species worldwide (Lanini and Kogan, 2005). Although cultivated tomatoes are usually susceptible (Ashton, 1976), some specific Heinz tomato cultivars that are resistant to *Cuscuta* spp. were discovered in the field (Hembree et al., 1999; Goldwasser et al., 2001). These resistant cultivars have been used in the field to control the infestation of *Cuscuta* spp., but the resistance mechanism remains unknown. Therefore, to identify the underlying mechanism and genes involved in these defense responses, these dodder-resistant Heinz tomatoes were used for further study. We discovered that the resistance response in these Heinz cultivars is based on post-attachment lignification in the stem cortex upon *C. campestris* infection. Recent work described the involvement of lignin in the resistance responses to root parasitic plants, including *Orobanche cumana*, *Orobanche minor*, *Phtheirospermum japonicum*, and *Striga hermonthica* (Labrousse et al., 2001; Kusumoto et al., 2007; Cui et al., 2018). However, considering the differences in the anatomy of stems and roots, whether host plants deploy similar mechanisms to stop stem parasitic plants remains under-investigated. Based on our comparative transcriptomics, virus-based gene expression studies in susceptible cultivars, and virus-induced gene silencing in resistant cultivars, we propose two transcription factors (TFs), *SlMYB55* (Solyc10g044680) and *Lignin induction Factor 1* (*LIF1*, an *AP2*-like protein) (Solyc02g079020), which regulate the biosynthesis of lignin in the cortex. Moreover, *Cuscuta R-gene for Lignin-based Resistance 1* (*CuRLR1*) (Solyc04g009110, a *CC-NBS-LRR*) may be engaged in signaling or function as a receptor for perceiving *C. campestris* signals or effectors, leading to lignification-based resistance. The overexpression of *CuRLR1* in susceptible tomato only induced strong lignification upon *C. campestris* attachment or *C. campestris* extract injection. To investigate whether these lignin-based resistance responses connect with previously identified *CuRe1-*mediated resistance responses, we conducted comprehensive RNA sequencing (RNA-seq) profiling, clustering, and gene coexpression analysis. Our gene co-expression networks (GCNs) indicate that *CuRe1* is also connected with *CuRLR1*, *LIF1*, and *SlMYB55* in resistant cultivars under *C. campestris* infested condition and also helped us identify another TF, *SlWRKY16* (Solyc07g056280), which has a similar expression pattern as *CuRe1*. Clustered regularly interspaced short palindromic repeats (CRISPR)-mediated mutants of *SlWRKY16* showed lignification in the cortex and were more resistant to *C. campestris*. This result indicates that *SlWRKY16* functions as a negative regulator of the lignin-based resistance. Furthermore, we noticed that the lignin-based resistance responds to a large protein molecule from *C. campestris* extracts, which might represent previously unidentified *C. campestris* signals or effectors. In summary, we discovered four key regulators control the lignin-based resistance response in the stem cortex upon *C. campestris* infection, and this lignification blocks *C. campestris* strands from parasitizing selected Heinz tomato cultivars.

## Results

### Response to *C. campestris* in the resistant cultivars

**Figure 1:**
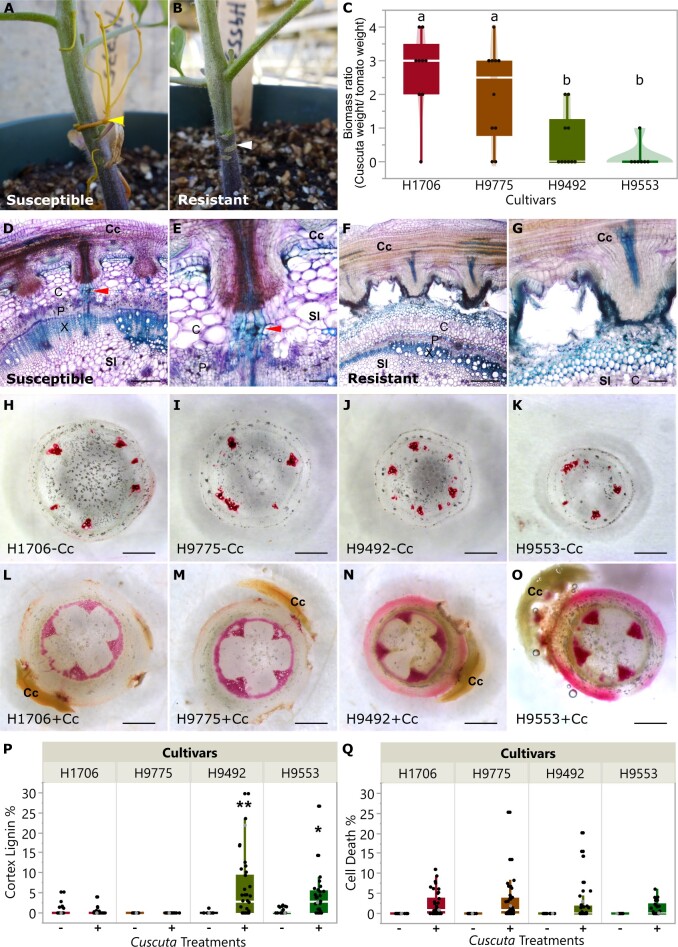
The comparison of resistance responses to *C. campestris* in tomato cultivars. (A) *Cuscuta campestris* grows on the susceptible H9775, (B) and cannot attach on the resistant H9553. Yellow arrowhead indicates *C. campestris* formed successful haustorial attachment. White arrowhead indicates *C. campestris* haustoria detached and left the scar on the host stem. C, The biomass ratio of host and *C. campestris* (*Cuscuta* weight/tomato weight) on different cultivars. Data were assessed using pairwise comparisons with Tukey’s test. Different letters indicate these groups are statistically significant different. *P*-values between “a” and “b” are <0.05. H1706, *n* = 9; H9775, *n* = 10, H9492, *n* = 10, H9553 *n* = 7. Data were collected at 45 DPA. D–G, 100-μm vibratome longitudinal sections of *C. campestris* haustoria attaching to H1706 (D and E) and H9553 (F and G), and stained with Toluidine Blue O. Lignin is stained as blue. Red arrowhead indicates haustorial vascular connections. Cc indicates *C. campestris*; Sl indicates *S. lycopersicum.* C, cortex; P, phloem; X, xylem. E and G are zoom-in image of the same section of D and F. D and F, Scale bar, 40 µm. E and G, Scale bar, 10 µm. (H–O) are ∼300 μm sections of the haustoria attachment sites stained with Ph–HCl. Scale bar, 1 mm. Lignin is stained as red. Stem cross-sections of H1706 (H and L), H9775 (I and M), H9492 (J and N), and H9553 (K and O) without *C. campestris* treatment (labeled with −Cc) and with *C. campestris* attached (labeled with +Cc). P, Cortex lignin area percentage in different cultivars. Data presented are assessed using multiple comparisons with Dunnett’s test. *P< 0.05, **P < 0.01. Q, Cell death area percentage in different cultivars. P and Q, H1706-Cc, *n* = 20; H1706+Cc, *n* = 38; H9775−Cc, *n* = 19; H9775+Cc, *n* = 40; H9492−Cc, *n* = 16; H9492+Cc, *n* = 38; H9553−Cc, *n* = 17; H9553+Cc, *n* = 30. Data were collected at 14 DPA. The data points labeled with gray color indicate the sample that we show in the section picture (H–I). C, P, and Q, The boxplot consists of a box extending from the 25th quantile to the 75th quantile. The centerline in the box indicates the median. The length of the box is the interquartile range (IQR), which is the difference between the 25th quantile and the 75th quantile. The whiskers extend from the ends of the box to the outermost data point that falls within 1.5 times of IQR. Points outside of the whiskers are outliers.

While most tomato cultivars can be parasitized by *C. campestris*, the Heinz hybrids 9492 and 9553 (H9492 and H9553) exhibit resistance to dodders (Goldwasser et al., 2001). *Cuscuta* *campestris* strands grew well on the susceptible H1706 (genome sequenced) and H9775 (Heinz hybrid 9775—closely related to the resistant cultivars; Figure 1A). On the other hand, *C. campestris* strands could not form good attachments with H9492 and H9553, and haustoria detached from the host stem, preventing parasite growth (Figure 1B). Based on the biomass ratio of *C. campestris* and host (relative growth rate), H9492 and H9553 cannot support long-term (>45 days) growth of *C. campestris*, in contrast to H9775 and H1706 (Figure 1C).

To identify the basis for resistance, we analyzed *C. campestris* attachments on susceptible and resistant lines using anatomy and cell wall-specific staining with Toluidine Blue O and phloroglucinol–HCl (Ph–HCl; Liljegren, 2010). Upon challenging these different cultivars with *C. campestris* strands, lignin accumulation in the stem cortex was observed in the resistant H9492 and H9553, but not in the susceptible H9775 and H1706 (Figure 1, D–O). The resistance mechanism involved local lignification in the stem cortex, creating a barrier to haustorium penetration, and dodder attachment on the resistant cultivars (Figure 1, D–O). Little to no lignin accumulates in the cortex of both resistant and susceptible cultivars without *Cuscuta* attachment (Figure 1P). In addition, *Cuscuta* attachment sites usually cause some wounding responses and cell death in both resistant and susceptible cultivars (Figure 1Q).

### Identifying the key time point for early host defense responses in host–parasite interactions

Changes in the levels of salicylic acid and jasmonic acid have been reported at 36–48 h after attachment (Runyon et al., 2010). To capture the earliest responses to dodder parasitism, we performed a time-course RNA-seq analysis on 0, 1, 2, 3, and 4 d post attachment (DPA) of *C. campestris* on tomatoes H1706 (susceptible). At these stages, the dodder strands were not embedded in the host and could be removed to collect the attached stem area. Within the constraints of our ability to identify differentially expressed genes (DEGs), which can be prone to both biological and technical variation, maximal transcriptional changes peaked at 4 DPA (Supplemental Figure S1 and Supplemental Data Set S1), suggesting that the DEGs include core genes involved in the early response to *C. campestris* infection. Accordingly, we chose 4 DPA for further gene expression analysis of the resistant and susceptible cultivars.

### Gene expression in the resistant and susceptible host response to *C. campestris*

We challenged the resistant H9492 and H9553, and susceptible H9775 and H1706 with *C. campestris* strands. We collected stem tissues at 4 DPA for RNA-seq and differential gene expression (DGE) analysis in dodder infested versus uninfested plants. In principal component analysis (PCA) on the transcriptomes of resistant and susceptible cultivars (Supplemental Figure S2), PC1 accounted for 44% of the variation and substantially clustered the data into two separate sets: infested and noninfested samples. However, PCA did not separate different cultivars into distinct genotypic groups. Thus, the transcriptional differences in response to *C. campestris* between the resistant and susceptible genotypes likely involve a small number of genes.

**Figure 2:**
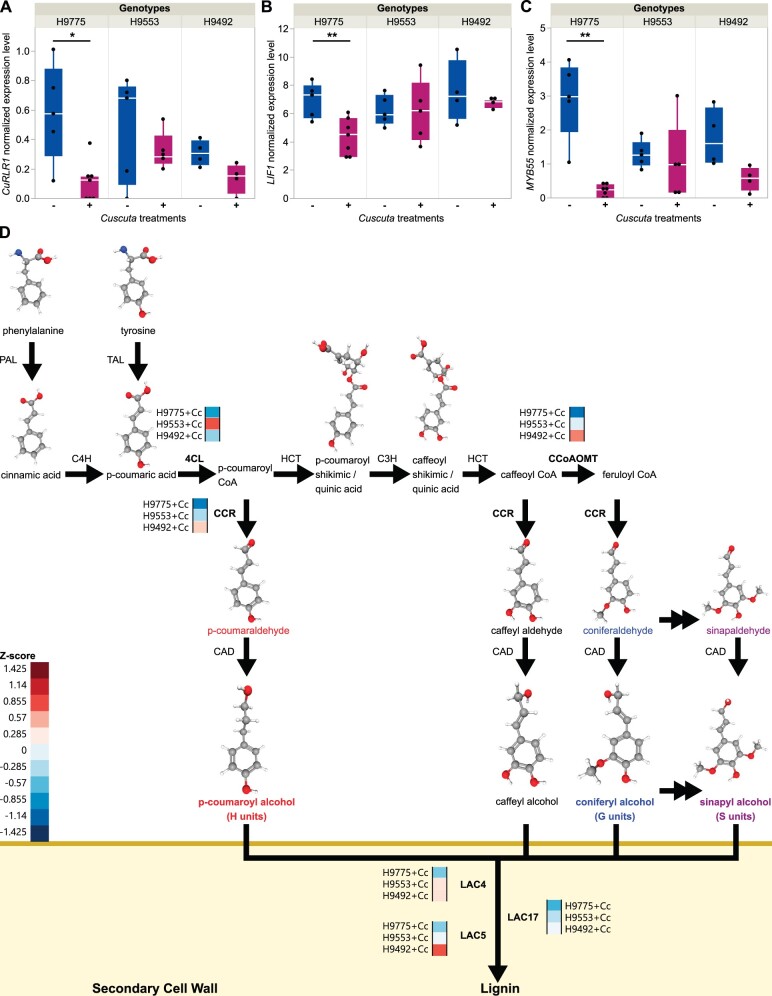
Key candidate genes and lignin biosynthesis genes that display expression changes upon *C. campestris* infestation. A–C, The normalized expressions levels (CPM, counts per million) of genes in susceptible cultivar H9775 and in resistant hybrid cultivar H9553 and H9492 under *C. campestris* infestation. – and + indicates without or with *C. campestris* infection treatments, respectively. Biologically independent replicates: RNA-seq libraries: H9775−Cc, *n* = 5; H9775+Cc, *n* = 7; H9492−Cc, *n* = 4; H9492+Cc, *n* = 4; H9553−Cc, *n* = 5; H9553+Cc, *n* = 5. Data are assessed using two-tailed *t* test. *P < 0.04, **P < 0.01, ***P < 0.005. The boxplot consists of a box extending from the 25th quantile to the 75th quantile. The centerline in the box indicates the median. The length of the box is the IQR, which is the difference between the 25th quantile and the 75th quantile. The whiskers extend from the ends of the box to the outermost data point that falls within 1.5 times of IQR. Points outside of the whiskers are outliers. D, The lignin biosynthesis pathway with key enzyme expression levels. Genes that are differentially expressed were selected and the normalized expression values across three cultivars were color coded according to *z*-score. + Cc indicates with *C. campestris* infection treatments. TAL, tyrosine ammonia-lyase; 4CL, 4-coumarate CoA ligase; HCT, hydroxycinnamoyl-CoA shikimate/Quinate hydroxycinnamoyltransferase; C3H, *p*-coumarate 3-hydroxylase; CCoAOMT, caffeoyl-CoA O-methyltransferase; CCR, cinnamoyl-CoA reductase; CAD, cinnamyl alcohol dehydrogenase; LAC, laccase. 3D structure images of phenylalanine, tyrosine, cinnamic acid, p-coumaric acid, p-coumaroyl shikimic acid, caffeoyl shikimic acid, *p*-coumaraldehyde, *p*-coumaryl alcohol, caffeyl aldehyde, caffeyl alcohol, coniferaldehyde, coniferyl alcohol, sinapaldehyde, and sinapyl alcohol are from PubChem (National Center for Biotechnology Information, 2021a, 2021b, 2021c, 2021d, 2021e, 2021f, 2021g, 2021h, 2021i, 2021j, 2021k, 2021l, 2021m, 2021n).

Next, we conducted DGE analyses by comparing *C. campestris* infested and uninfested host plants using an interaction design model (design model = infested or uninfested condition + genotype + condition: genotype). Based on our communication with the Kraft Heinz Company, both H9492 and H9553 were developed in the same breeding program. However, H9553 is more resistant to *C. campestris* than H9492 based on the relative growth rate results (Figure 1C). Therefore, we suspected that the enhanced resistance to *C. campestris* is due to alterations in key regulatory gene expression or function. We selected 113 genes that were differentially expressed (Supplemental Data Set S2) between infested H9775 (susceptible) and infested H9553 (resistant) with an adjusted *P*-value cutoff < 0.1 and log2 fold change >1. Consistent with our observations of lignin accumulation in resistant tomato cultivars upon *C. campestris* infestation (Figure 1), many of these genes are known to be involved in the lignin biosynthetic pathway, including three laccase genes (*LAC4*, *5*, and *17*, Solyc05g052340, Solyc09g010990, Solyc10g085090) and Caffeoyl CoA 3-O-methyltransferase (*CCoAOMT*, Solyc01g107910; Figure 2).

However, many of the genes with low adjusted p values among these 113 DGEs are genes with unknown functions, or are genes involved in downstream functions, like enzymes or transporters. To narrow down the potential candidates and identify upstream master regulators controlling this lignin-based resistance, we focused on TFs (based on gene annotations) as possible key regulators of lignin biosynthesis pathways, and membrane-localized or cytosolic receptors that may receive signals from *C. campestris*. Using these two criteria, we identified three candidate genes for further study, including a TF related to *AP2*, a *SlMYB55* TF, and a gene encoding an N-terminal coiled-coil nucleotide-binding site leucine-rich repeat protein (*CC-NBS-LRR*; Figure 2, A–C). Among the 113 DEGs, Solyc04g009110 was the only *CC-NBS-LRR* gene and there are only six TFs in the list. Among these TFs, *MYB*, *WRKY*, and *Ethylene-responsive transcription factor* gene families are often reported to be involved in regulating general lignin biosynthesis or lignin accumulation upon pathogen infection in many other plant organisms (Zhong and Ye, 2009; Ma et al., 2017; Xie et al., 2018; Miyamoto et al., 2020). Therefore, among these TF gene families, we selected *SlMYB55* (Solyc10g044680), which ranked as the first TF based on multiple comparison-adjusted *P*-values, for further analysis. However, we were not able to find any reports on *AP2/B3* domain-containing protein being involved in lignin biosynthesis. Therefore, we suspected that this TF might play an as yet undescribed role in the lignin-based resistant response against *C. campestris* outside of general lignin biosynthesis or general defense responses. Hence, we selected this *AP2/B3* domain-containing protein (Solyc02g079020), which ranked as the fourth TF based on multiple comparison-adjusted *P*-values, as one candidate gene as well. These three candidate genes were identified in our infested H9775 (susceptible) and infested H9553 (resistant) RNAseq analysis as differentially expressed and sharing a common expression pattern of significantly reduced expression levels upon *C. campestris* infestation in the susceptible cultivars. However, expression of these three candidate genes remained almost unchanged from uninfested or was only mildly reduced upon *C. campestris* infestation in resistant cultivars. This result suggests that these candidates might play a role in defense against *Cuscuta*, such that when these genes are not repressed during *C. campestris* infestation, the host plants are more resistant to *C. campestris*.

### Functional characterization of candidate genes using virus-based gene expression and virus-induced gene silencing

**Figure 3:**
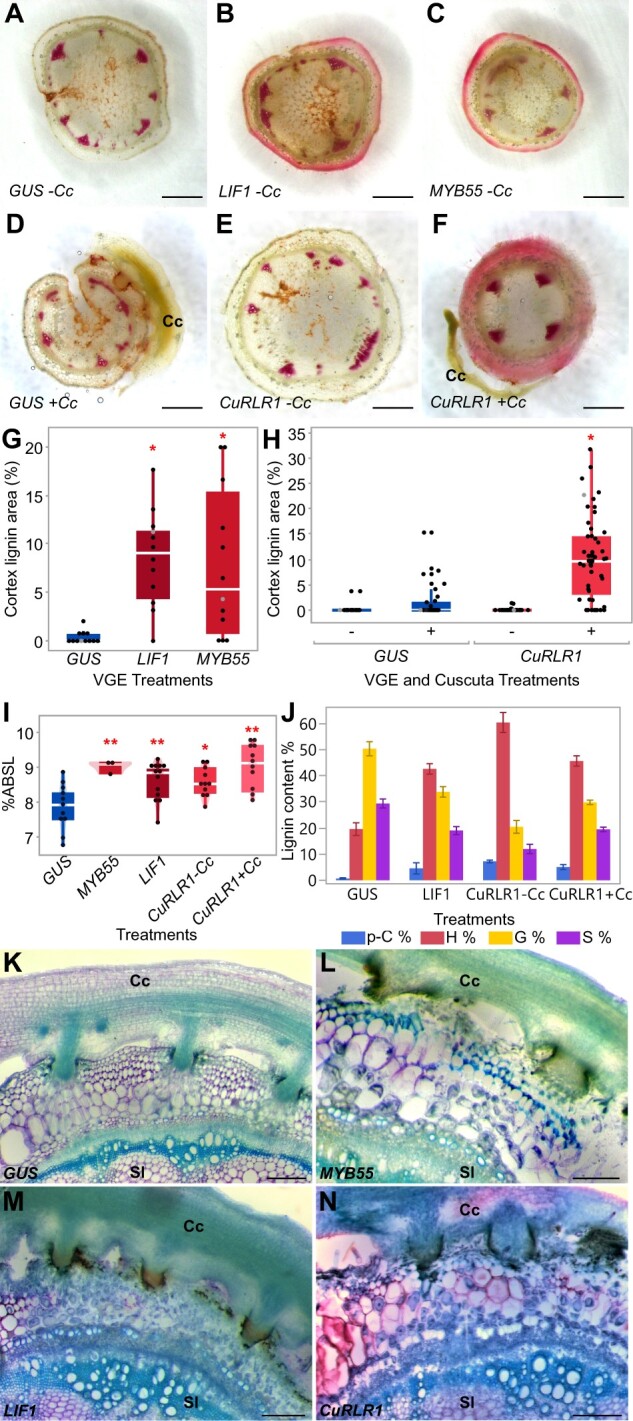
VGE in tomato H1706. A–F, ∼300-μm stem sections near injection sites, Ph–HCl stains lignin red. Scale bar, 1 mm. VGE of *GUS* (A), *LIF1* (B), *SlMYB55* (C), and *CuRLR1* (E) in stem without *C. campestris*. VGE of *GUS* (D) and *CuRLR1* (F) with *C. campestris*. G, Cortex lignin area percentage in VGE of *LIF1* and *SlMYB55* (*n* = 12 each) and H, *CuRLR1* with and without *C. campestris* (*GUS*-Cc, *n* = 18; *GUS*+Cc, *n* = 28; *CuRLR1*−Cc, *n* = 47; *CuRLR1*+Cc, *n* = 53). G and H, Data were collected at 7-d post injection (DPI) and 14 DPA. Data are assessed using Dunnett’s test with *GUS*−Cc as negative control. *P < 0.01. The data points labeled with grey color indicate the sample that we show in the section picture (A–F). I, Acetyl bromide assay for lignin in VGE stems. Acetyl bromide soluble lignin (ABSL) indicates percent absorbance of soluble lignin. Samples were collected at 7 DPI and 6 DPA. Data are assessed using Dunnett’s test. *P <0.05, **P <0.01. Biological replicates for *GUS*, *n* = 18; *SlMYB55*, *n* = 10; *LIF1*, *n* = 18, *CuRLR1*-Cc, *n* = 18; *CuRLR1*+Cc, *n* = 18. Technical replicates for acetyl bromide assay; *GUS*, *n* = 11; *SlMYB55*, *n* = 3; *LIF1*, *n* = 13; *CuRLR1*−Cc, *n* = 11; *CuRLR1*+Cc, *n* = 11. G–I, The boxplot consists of a box extending from the 25th quantile to the 75th quantile. The centerline in the box indicates the median. The length of the box is the IQR, which is the difference between the 25th quantile and the 75th quantile. The whiskers extend from the ends of the box to the outermost data point that falls within 1.5 times of IQR. Points outside of the whiskers are outliers. J, Pyro-GC assay for monolignols in *CuRLR1* VGE samples with and without *C. campestris*. p-C, p-coumaric acid; H, H types of (p-coumaryl alcohol); G, G types of monolignol (coniferyl alcohol); S, S types of monolignol (sinapyl alcohol). Samples were collected at 7 DPI and 6 DPA. Biological replicates collected from first internodes; *GUS*, *n* = 8; *LIF1*, *n* = 8, *CuRLR1*-Cc, *n* = 18; *CuRLR1*+Cc, *n* = 18. Pyro-GC assay technical replicates; *GUS*, *n* = 3; *LIF1*, *n* = 3; *CuRLR1*-Cc, *n* = 5; *CuRLR1*+Cc, *n* = 5. K–N, VGE of *CuRLR1*, *LIF1* and *SlMYB55* induces cortical lignin making H1706 resistant to *C. campestris*. Scale bar, 30 µm. Samples were collected at 7 DPI and 6 DPA. Cc indicates *C. campestris*; Sl indicates *S. lycopersicum.*

To validate the function of any candidate genes, an ideal method is to generate knockout mutant plants for further study. However, these resistant tomato lines are F1 hybrids in the Heinz background, and the Heinz cultivars are recalcitrant to transformation. Further, the lignin-based phenotype in the resistant cultivars is accompanied with loss of downregulation of the candidate genes upon Cuscuta infestation. To evaluate if the candidate genes can confer lignification-based resistance in susceptible tomato cultivars, we cloned coding regions of *GUS, AP2*-like, *SlMYB55*, and *CC-NBS-LRR* genes into virus-based gene expression (VGE) vectors (vector map in Supplemental Figure S3; sequence in Supplemental Data Set S3) for transient expression in the susceptible H1706, which has similar expression patterns of these three candidate genes (Supplemental Figure S4, A–C). We saw substantial GUS expression in the stem around the injection site (Supplemental Figure S4, D–F), and a lack of lignification due to the process of injection itself (Figure 3, A and D). Hence, we used *GUS*-injected plants as our mock controls for VGE experiments. We sectioned and stained injected stems with lignin-specific Ph–HCl for lignin detection. VGE with *SlMYB55* and *AP2*-like successfully overexpressed these genes in the first internode near the injection site and induced stem lignification in the susceptible H1706 without *C. campestris* infestation (Figure 3, B, C, and G). Therefore, we named this *AP2*-like protein *LIF1* (Solyc02g079020) based on its ability to induce lignin biosynthesis in the cortex. These results indicate that *SlMYB55* and *LIF1* might play a role in regulating some of the critical enzymes in the lignin biosynthesis pathway.

In contrast, the H1706 plants with VGE of *CC-NBS-LRR* had no lignin accumulation phenotype and were very similar to those with GUS VGE under no *C. campestris* infestation conditions (Figure 3E). However, previous studies indicated that many genes in the *NBS-LRR* family encode intracellular receptors that detect pathogens and trigger defense signaling (Padmanabhan et al., 2013). Therefore, we suspected that this *CC-NBS-LRR* might play a role in signaling or function as a receptor for signals from *Cuscuta* that are needed to initiate subsequent defense responses. Hence, *C. campestris* infestation treatment might be needed to see the phenotype difference. To validate this hypothesis, we compared the response differences between *Cuscuta* infested and uninfested susceptible H1706 with *CC-NBS-LRR* VGE (Figure 3, E,F, and H). Intriguingly, our results showed the expression of *CC-NBS-LRR* only induced lignification upon *C. campestris* attachment (Figure 3H), and these results suggest that direct or indirect perception of *C. campestris* signals by this *CC-NBS-LRR* leads to lignification-based resistance. Thus, we named this gene (*CuRLR1*.

To further bolster our findings, we used virus-induced gene silencing (VIGS) to knock down our candidate genes in the resistant cultivar H9553 to test their function. The most successful VIGS technology involves downregulation of candidate genes that lead to a visible phenotype and these phenotypes are often inconsistent (Orzaez et al., 2009). In our system, we are looking at phenotypes that can manifest when two organisms interact with each other, and the VIGS system is not robust enough to capture these interactions consistently. Here we show that the *C. campestris* plants grown on *AP2*-like, *SlMYB55*, and *CC-NBS-LRR* VIGS knockdown plants have higher survival rates compared with those growing on mock controls (Supplemental Figure S5). Similar phenotypes were also observed in *CCoAOMT* (Solyc01g107910) and *LAC* genes (*LAC4*, *5*, *17*, three genes in one construct (Solyc05g052340, Solyc09g010990, Solyc10g085090)) knockdown plants (Supplemental Figure S5). These results indicate that these candidate genes might play a role in the lignin-based resistance response. Therefore, when these essential genes were silenced by VIGS in resistant tomato cultivars, resistant tomato became more susceptible to *C. campestris*, leading to a higher survival rate of the parasite.

We are also aware that lignin is a complex polymer and Ph–HCl staining is a fast and efficient lignin detection method, but it only detects the cinnamaldehyde end groups of lignin, preferentially staining the G and S-type aldehyde form monolignols (Pomar et al., 2002; Cass et al., 2015). Therefore, we also conducted an acetyl bromide assay to determine total lignin content, including different types of monolignols and lignin precursors. Consistent with the aforementioned anatomical observations, the overexpression of *SlMYB55* and *LIF1* both increased total lignin content compared with GUS mock controls in this assay (Figure 3I). Surprisingly, the overexpression of *CuRLR1* also increased the total lignin content even without *Cuscuta* signals. With *Cuscuta* signals, the total lignin content was much higher in *CuRLR1* overexpressing plants (Figure 3I). This difference indicates that the composition of induced lignin might be different between *CuRLR1* overexpressing plants with and without *Cuscuta* signals.

To further validate this hypothesis, we used high-performance liquid chromatography (HPLC) and pyrolysis gas chromatography/mass spectrometry (pyro-GC-MS) to analyze the composition of induced lignin. Our HPLC results showed that *p*-coumarate and trans-ferulate are both increased in *CuRLR1* overexpressed plants, but the samples with *Cuscuta* signals have much higher levels of these two precursors than the samples without *Cuscuta* signals (Supplemental Figure S6). Pyro-GC-MS analysis showed that samples from *CuRLR1* overexpressing plants without *Cuscuta* signals have the larger percentage of H-lignin and the larger concentration of coumarate derivatives compared to VGE of *GUS*, *LIF1*, and *CuRLR1* with *Cuscuta* (Figure 3J). These results show that *CuRLR1* overexpression alone leads to an increase in the upstream steps of the lignin biosynthesis pathway and production of more lignin precursors and H-type monolignols, while adding *Cuscuta* signals may actually upregulate the final steps in the biosynthesis pathway leading to more G-type and S-type monolignol formation (Figure 3, I and J). Since H-lignin and coumarate are not incorporated into lignin as aldehydes, they are not detected by phloroglucinol staining, which explains the difference that we observed between the phloroglucinol staining data and acetyl bromide assay. *CuRLR1* overexpression alone induced the accumulation of lignin precursors and H-lignin, which might function as a baseline defense response. Based on previous studies, H-lignin has been associated with both stress response as well as defense from pathogen intrusion because this is a form of “defense” lignin that can be generated and deposited more rapidly than G or S lignin (Zhang et al., 2007; Moura et al., 2010; Liu et al., 2018). Upon detecting *Cuscuta* signals, resistant tomatoes accumulate more G-type and S-type monolignols to strengthen the baseline defense response and reinforce a stronger physical boundary.

Eventually, whether or not the overexpression of these candidate genes makes susceptible tomatoes resistant to *C. campestris* is the central question when evaluating potential agricultural applications. Therefore, we transiently overexpressed *SlMYB55*, *LIF1*, and *CuRLR1* first and then attached *C. campestris* strands to test their resistance status. Based on our results, VGE of *SlMYB55*, *LIF1*, and *CuRLR1* with *C. campestris* all induced lignin accumulation in the cortex and blocked haustorium penetration, which made the susceptible tomato cultivar H1706 more resistant to *C. campestris* (Figure 3, K–N; Supplemental Figure S7 and Supplemental Data Set S4).

### Regulatory mechanisms and networks leading to resistance responses

Since both H9492 and H9553 hybrid cultivars arose in the same breeding program, enhanced resistance to dodders observed in these two cultivars is likely due to the presence of some unique sequence polymorphisms in these cultivars. Resistance-specific single-nucleotide polymorphisms (SNPs) could contribute to the regulation or function of our candidate genes, so we specifically identified SNPs that are common in H9553 and H9492 but different from H9775 (Supplemental Data Set S5). Unexpectedly, there were no resistance-specific SNPs in coding regions of our candidate genes except one SNP located in a *LIF1* exon. This resistance-specific SNP changes 251 Lysine (K, in H1706) to 251 Glutamine (Q, in H9553). However, based on our protein domain prediction using InterProScan (Jones et al., 2014; Mitchell et al., 2018) and protein structure analysis using Phyre2 (Kelley et al., 2015), this amino acid replacement is not located in any known protein domains or structures. We also conducted Protein Variation Effect Analyzer (PROVEAN) analysis, and this K251Q variant only has a 0.619 PROVEAN score, indicating that it is a neutral variant. Thus, based on our analysis, the K251Q variant seems unlikely to influence normal LIF1 protein function. Hence, we suspect that transcriptional regulation or protein–protein interactions might be key regulatory mechanisms of resistance.

We, therefore, specifically focused on resistance-specific SNPs in the promoter regions of our candidate genes (Supplemental Data Set S6). Our SNP analysis detected several resistance-specific SNPs in the *LIF1* promoter region, but no resistance-specific SNPs were detected in other candidate gene promoter regions (within 5-kb upstream). Furthermore, since CuRe1 was the first *Cuscuta* signal receptor identified in tomatoes, we hypothesized that the candidate genes that we identify could also have crosstalk with the CuRe1 signaling pathway. Therefore, we included *CuRe1* in our data set for further analysis. One resistance-specific SNP was detected in the *CuRe1* promoter region (outside 5-kb upstream) located at a putative YABBY binding site. However, this SNP is also located 1,184-bp upstream of *UBIQUITIN-LIKE PROTEASE1* (*ULP1*; Solyc08g016275) and may regulate expression of this neighboring gene instead of *CuRe1*. Therefore, we focused on the *LIF1* promoter region for further analysis and conducted TF binding site predictions.

Based on our phylogenetic network analysis (Solís-Lemus et al., 2017) using 500 kb around the *LIF1* resistance-specific SNP-enriched region, these SNPs might be introgressed from wild tomato species (likely coming from *S. galapagense* and/or *S. pennellii*, Supplemental Figure S8). One of these resistance-specific SNPs is located right at a WRKY binding W-box cis-element (TTGACY-core motif; Ciolkowski et al., 2008; Chen et al., 2019; Supplemental Figure S9 and Supplemental Data Set S7). This SNP is predicted to interrupt WRKY binding, potentially leading to *LIF1* expression differences between resistant and susceptible cultivars upon *C. campestris* attachment. Hence, we were also interested in searching for potential *WRKY* TFs in our selected gene lists.

**Figure 4:**
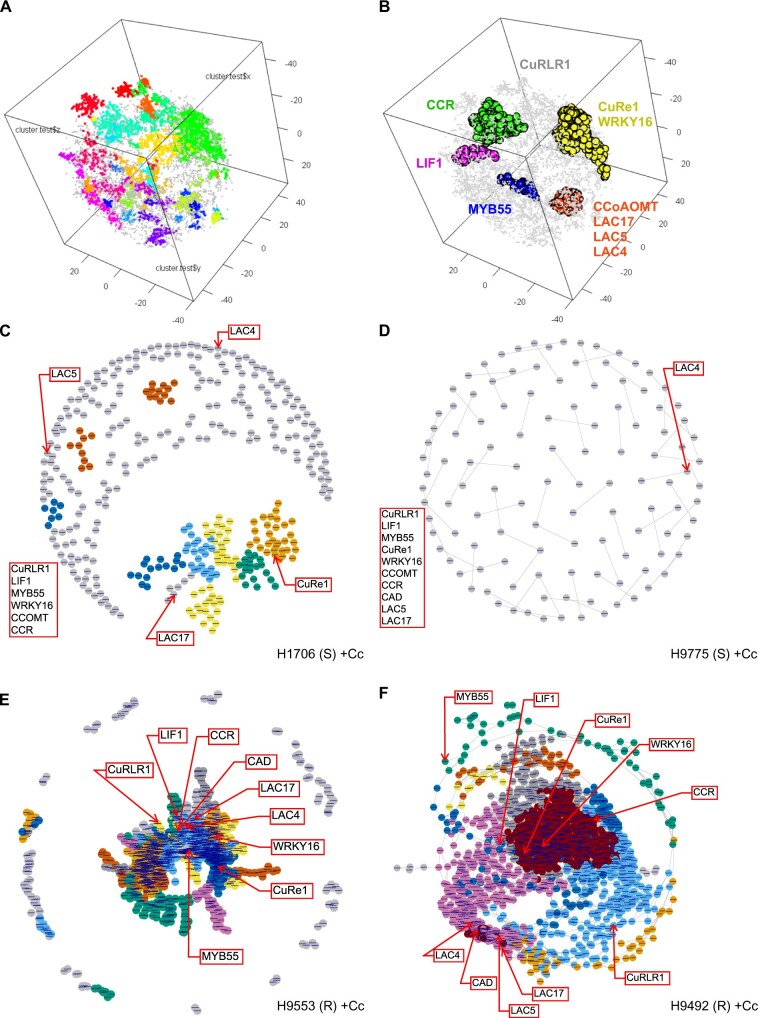
BH t-SNE generated gene clusters and GCN analysis. A, BH t-SNE generated gene clusters based on their gene expression patterns. Total cluster (module) number was 48. Different colors indicate different clusters. B, The candidate genes that are included in the clusters are labeled with their corresponding colors. The selected gene clusters for GCN are labeled in yellow (CuRe1 and WRKY16 cluster, cluster 17), red (CCOMT and LAC cluster, cluster 11), blue (MYB55 cluster, cluster 39), pink (LIF1 cluster, cluster 46), green (CCR cluster, cluster 23) colors. *CuRLR1* is in the noise cluster, and is labeled in gray color. Parameters used in this analysis: perplexity (perp) = 20, lying = 250, cutoff = 20, seed = 2. C–F, GCNs of four different Heinz susceptible and resistant cultivars upon *C. campestris* treatments. S indicates susceptible; R indicates resistant. Based on BH t-SNE analysis, 1,676 genes in cluster 11, 17, 23, 39, 46, and CuRLR1 were selected for building GCNs. +Cc indicates with *C. campestris* infection treatments. Different colors of the nodes indicate different modules based on GCN community structure. The genes that are listed at the left of the GCN and not labeled in the network are the genes that have no coexpression connections with all the other genes in the list.

To understand the relationships between the three candidate genes and their targets, to identify the potential *WRKY* regulator, and also to investigate whether these candidate genes connect with previously identified *CuRe1*-mediated resistance responses (Hegenauer et al., 2016), we conducted DGE analysis on our resistant and susceptible tomato cultivar RNA-seq data with ANOVA and selected 10,939 DEGs with FDR <0.1 (Supplemental Data Set S8). Next, we used Barnes–Hut t-distributed stochastic neighbor embedding (BH t-SNE) to generate gene clusters using RSMod (a pipeline developed by us, script included in code availability) (Ranjan et al., 2016). In this analysis, 5,941 DEGs are clustered into 48 groups based on their gene expression patterns and 4,998 DEGs are in the noise group. Among the 48 gene clusters generated (Supplemental Data Set S9), five clusters were selected based on their gene ontology (GO) enrichment terms and the candidate genes they included (Figure 4, A and B; Supplemental Data Set S9). The GO term of cluster 39 is “DNA binding”, which includes potentially key resistance TFs, like *MYB55*. The GO term of cluster 11 is “lignin biosynthetic and catabolic process”, which encapsulates the observed resistance phenotypes, and includes Caffeoyl CoA 3-O-methyltransferase (*CCOMT*) and three Laccase (*Lac*) genes identified in our model-based approach (Supplemental Data Set S2). Cluster 23 includes a *Cinnamoyl-CoA reductase* gene (Solyc03g097170, *CCR*) and is enriched in the “xyloglucan:xyloglucosyl transferase activity” GO term, which may indicate potential cell wall modifications. “Response to biotic stimulus” is the GO term enriched in cluster 17, which also includes the previously identified Cuscuta receptor, *CuRe1*.

**Figure 5:**
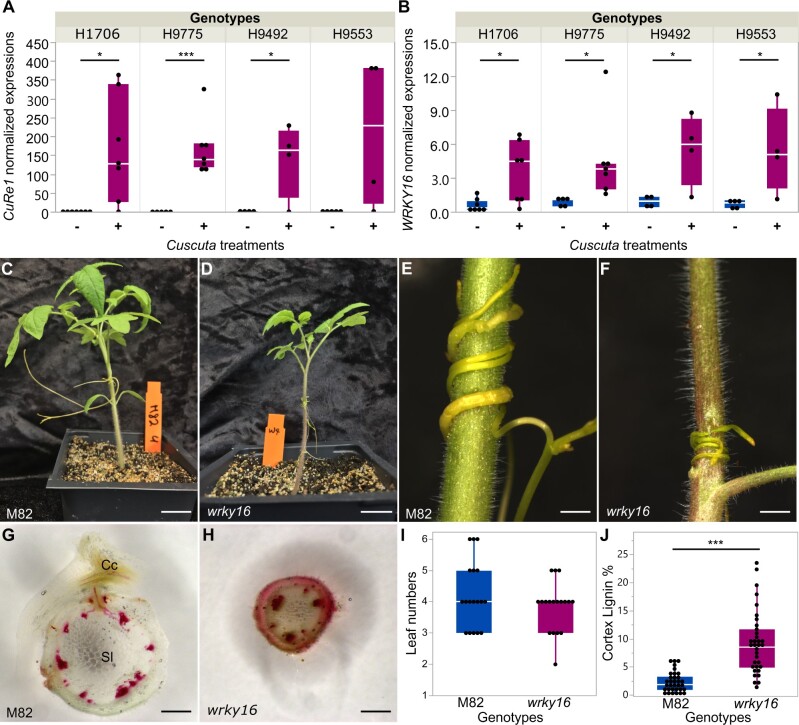
The role of *SlWRKY16* in *Cuscuta* resistance using CRISPR/Cas9 gene knockouts. A and B, Normalized *CuRel* and *SlWRKY16* expression level from RNA-seq data (CPM, counts per million) in different Heinz cultivars with/without *Cuscuta* treatments. Biologically independent replicates: RNA-seq libraries, H1706-Cc, *n* = 7; H1706+Cc, *n* = 7; H9775−Cc, *n* = 5; H9775+Cc, *n* = 7; H9492−Cc, *n* = 5; H9492+Cc, *n* = 4; H9553−Cc, *n* = 5; H9553+Cc, *n* = 5. Data are assessed using one-tailed *t* test. *P <0.05, ***P <0.005. C–J, Samples and data were collected at 7 DPA. C and D, Overall phenotype comparison between M82 and homozygous *SlWRKY16* CRISPR lines (*wrky16*). Scale bar, 2 cm. E and F, *Cuscuta campestris* growing on M82 and *wrky16*. Scale bar, 2 mm. G and H, ∼300-μm hand sections of M82 and *wrky16* stems near *Cuscuta* attachment site stained with Ph–HCl. Lignin is stained red. Cc indicates *C. campestris*; Sl indicates *S. lycopersicum.* Scale bar, 1 mm. I, Leaf number of *wrky16* and M82. Biological replicates, *n* = 18 for each. J, Cortex lignin area percentage in M82 and *wrky16* stems. Data presented are assessed using Student’s *t* test. ***P<0.001. Replicates: M82, *n* = 33; *wrky16*, *n* = 34. A, B, I, and J, The boxplot consists of a box extending from the 25th quantile to the 75th quantile. The centerline in the box indicates the median. The length of the box is the IQR, which is the difference between the 25th quantile and the 75th quantile. The whiskers extend from the ends of the box to the outermost data point that falls within 1.5 times of IQR. Points outside of the whiskers are outliers.

Additionally, with comprehensive RNA-Seq clustering and gene coexpression analysis results, we also noticed *SlWRKY16* (Solyc07g056280) is always clustered with *CuRe1*. *SlWRKY16* was highly upregulated at 4 DPA in all four Heinz cultivars, an expression pattern similar to that for *CuRe1* (Figure 5, A and B). Host tissues surrounding haustoria from the tomato M82 cultivar also show upregulated expression of *SlWRKY16* at 4 DPA in our time-course data with FDR <0.1 and reverse transcription quantitative polymerase chain reaction (RT-qPCR) data (Supplemental Figure S10). Thus, *SlWRKY16* is a commonly upregulated host response gene across different cultivars and may play an important role in the transduction of *C. campestris* signals upon host attachment. Furthermore, one of the resistance-specific SNPs in the *LIF1* promoter region mentioned above, is located at a WRKY TF W-box (TTGACY-core motif) binding site, which is also the predicted *SlWRKY16* binding site based on homologous genes in the phylogenetic tree of the WRKY domain at the Plant Transcription Factor Database (Jin et al., 2016). Taking all these criteria together, we included *SlWRKY16* in our candidate genes for further analysis.

We focused on these 1,676 genes in clusters 11, 17, 23, 39, 46 and included *CuRLR1* (Figure 4, A and B; Supplemental Data Set S9) to construct GCNs for different treatments and cultivars to identify central hub genes (Figure 4; Supplemental Figure S11; script included in code availability). Interestingly, *CuRLR1*, *SlWRKY16*, and *CuRe1* had few connections or almost no connection with other genes in the GCN (with normal quantile cutoff = 0.997) in susceptible cultivars with *Cuscuta* attachments (Figure 4, C and D). On the other hand, *CuRe1* and *SlWRKY16* became central hub genes in resistant cultivars only upon *C. campestris* attachments and connected with *CuRLR1* (Figure 4, E and F). However, based on our DNA-Seq analysis, we cannot detect any resistance-specific SNPs in the promoter regions or coding regions of *CuRe1* and *CuRLR1*, and *SlWRKY16* (Supplemental Data Set S5). This result indicates that the differential expression of *CuRe1* and *CuRLR*1, and *SlWRKY16* may be controlled by trans-regulatory factors or protein interactions. Our GCN analysis and DNA-Seq analysis results show that all four Heinz tomato cultivars have the identical complete sequences of *CuRe1*, *CuRLR1*, and *SlWRKY16*. However, their expression patterns might be different depending on other trans-regulatory factors, which would require further investigation. Among them, *SlWRKY16* is a key factor in the transduction of *C. campestris* signals upon attachment of the parasite to the host. However, the differential expression patterns upon *C. campestris* attack and the diverse regulatory connections of these three genes determine whether resistance responses are triggered in these Heinz cultivars or not.

### Functional characterization of *SlWRKY16* by CRISPR/Cas9 knockouts and VGE

Since *SlWRKY16* exists in all Heinz resistant and susceptible tomatoes and in the M82 tomato cultivar, we bypassed the transformation limitation in Heinz tomatoes and generated mutant tomato plants in the M82 background for further analysis. To validate the function of *SlWRKY16* and its role in lignification-based resistance, we produced stable *SlWRKY16* edited M82 lines using the CRISPR/Cas9 targeted gene knockout system (Pan et al., 2016). Our homozygous null mutants were generally smaller than M82 wild-type (Figure 5, C and D) even though both *wrky16* and M82 wild type show the same developmental progression (Figure 5I). Intriguingly, *wrky16* plants are more resistant to *C. campestris* than M82 wild-type (Figure 5, E–H). Using Phloroglucinol staining, we noticed that homozygous *wrky16* lines continuously produce cortical lignin, which forms a physical boundary and provides a strong resistance to *C. campestris* attachment compared to M82 wild-type (Figure 5, E–H and J). However, the phenotype of continuously accumulating cortical lignin likely also limits cell growth and leads to the stunted growth phenotype in *wrky16* plants. Previous studies reported that plants with modified lignin content often showed changes in the balance of their growth-defense tradeoff, and some might have autoimmunity phenotypes (Ha et al., 2021). Therefore, we selected three known plant immunity marker genes, including *PATHOGENESIS-RELATED PROTEINS 1* (*PR1*), *DEHYDRATION-RESPONSIVE ELEMENT BINDING PROTEIN 1* (*DREB1*), and *DREB2*, to test if *wrky16* CRISPR plants have autoimmunity responses. Although *wrky16* plants slightly increase *PR1* expression, the expression changes in *PR1*, *DREB1*, and *DREB2* between wild-type M82 and *wrky16* are not statistically significant (Supplemental Figure S12). Therefore, the dwarf phenotype of *wrky16* is likely because of increased lignin content in stem cortical cell walls. These results indicate that *SlWRKY16* functions as a negative regulator of cortical lignin accumulation.

The hypothesis that *SlWRKY16* may play a role in the lignin-based resistance response also incorporates our previous SNP analysis and TF binding site prediction results in the *LIF1* promoter region (Supplemental Figure S9). We proposed that the resistance-specific SNP located at a WRKY binding site in the *LIF1* promoter region could interrupt SlWRKY16 protein binding, leading to *LIF1* expression differences between resistant and susceptible cultivars upon *C. campestris* attachment. Therefore, we conducted RT-qPCR to determine the expression levels of *LIF1* in both susceptible M82 wild-type and resistant *wrky16* tomatoes (M82 background). We observed a mild increase in *LIF1* expression in *wrky16* tomatoes compared to M82 tomatoes (Supplemental Figure S13A). Considering LIF1 is an AP2/B3-like TF, any elevation in a TF expression could potentially lead to large differences in the downstream gene expression pathways (Adcock and Caramori, 2009; Eguchi et al., 2016).

To evaluate the interaction between *SlWRKY16* and the other three candidate genes, we transiently overexpressed *LIF1*, *SlMYB55*, *CuRLR1*, and *GUS* controls in the susceptible H1706, M82 wild-type, and resistant *wrky16* tomatoes. In the *GUS* transient overexpression control group, we observed that *wrky16* plants accumulate much more lignin than H1706 and M82 wild-type as expected (Supplemental Figure S13B). The overexpression of *LIF1* induced more lignification in H1706, M82, and *wrky16* plants. This result shows additive effects of the loss of *SlWRKY16* function and overexpression of *LIF1* in lignification responses (Supplemental Figure S13B), suggesting that *SlWRKY16* may not only regulate *LIF1* expression at the transcriptional level, but also may regulate LIF1 protein function by other mechanisms, including protein–protein interactions. Also, the overexpression of *MYB55* induced more lignification in H1706 and *wrky16* plants (M82 background) but not in M82 (Supplemental Figure S13B), indicating that the loss of *SlWRKY16* function in M82 allows more lignin accumulation upon *MYB55* overexpression. This result also suggests subtle differences in resistant response between cultivars and that *SlWRKY16* might act upstream of *MYB55*, but more details remain to be elucidated in future research.

On the other hand, the overexpression of *CuRLR1* with *C. campestris* infection was able to induce more lignification in M82, but not in *wrky16* tomatoes (Supplemental Figure S14). This epistatic phenotype suggests that either *CuRLR1* or *SlWRKY16* are in the same pathway with WRKY16 downstream of CuRLR1, or that *CuRLR1* and *SlWRKY16* are in two independent pathways that may influence each other. This hypothesis matches with the GCNs we built, which show that *CuRLR1* and *SlWRKY16* are peripherally positioned in resistant cultivars in the *Cuscuta* treated condition, with multiple layers of genes connecting them. Furthermore, previous studies also indicated that WRKY family proteins could physically interact with other regulatory proteins to control several important biological processes (Chi et al., 2013). In order to elucidate other layers of regulation between these genes, we conducted protein–protein interaction investigations.

### Subcellular localization and interactions between the candidate proteins

**Figure 6:**
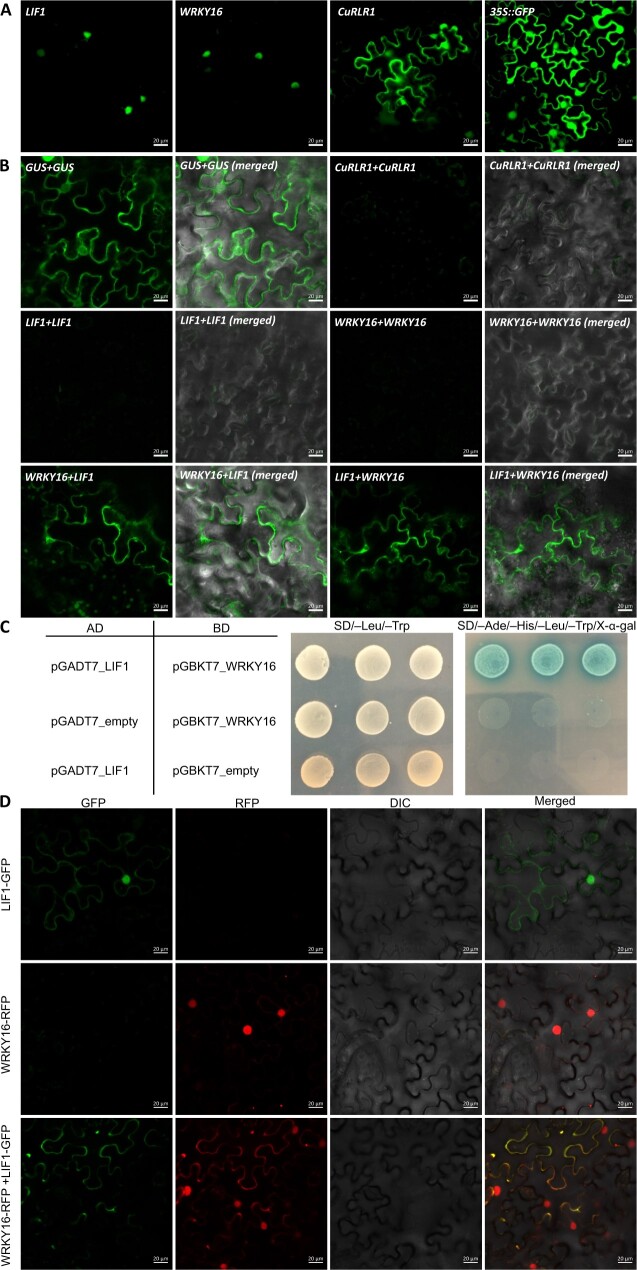
Subcellular localization of candidate genes and protein–protein interactions. A, Subcellular localizations of LIF1, SlWRKY16, and CuRLR1 proteins. B, Verification of protein–protein interactions and locations of SlWRKY16 and LIF1 by BiFC. The gene with *cCitrine* fusion is listed before the “+” sign and the gene with *nCitrine* fusion is listed after the “+” sign. C, Yeast two-hybrid (Y2H) results for interaction between LIF1 and SlWRKY16. The plasmids with GAL4 activation domain (AD) and GAL4 DNA binding domain (BD) were co-transformed to yeast AH109 competent cells. Transformed yeast cells were screened on SD/–Leu/–Trp medium plates to select successful co-transformants and then assayed by culturing on high-stringency SD/–Ade/–His/–Leu/–Trp medium plates with 40 μg·mL^−1^ X-α-Gal. The positive protein–protein interactions between LIF1 and SlWRKY16 are indicated by growth on SD/–Ade/–His/–Leu/–Trp/X-α-Gal medium plates and blue colony color. D, Co-expression of fusion protein LIF1-GFP and SlWRKY16-RFP to observe subcellular localizations. Yellow color in the merged panel indicates that GFP and RFP signals are overlapped. Scale bars for A, B, D: 20 µm.

One described mechanism for triggering innate immunity following TMV infection in *Nicotiana benthamiana* involved interaction and subsequent nuclear localization of the SPL6 TF with the TIR-NBS-LRR receptor (Padmanabhan et al., 2013; Padmanabhan and Dinesh-Kumar, 2014). Therefore, we investigated the potential interactions between our candidates and their protein subcellular localization to uncover potential regulatory mechanisms. Based on our results using translational GFP fusions, LIF1 and SlWRKY16 are located mainly in the nucleus (Figure 6A), while *CuRLR1* is located in both the nucleus and the cytosol. Bimolecular fluorescence complementation (BiFC) experiments with split YFP using transient infiltration in *N. benthamiana* leaves show that the LIF1 and SlWRKY16 proteins interact and get localized to the cytoplasm (Figure 6B). Interactions between other combinations, CuRLR1-LIF1, CuRLR1-SlWRKY16, or CuRLR1-CuRe1, were not detected in our experiments (Figure 6B).

To further validate the interaction between LIF1 and SlWRKY16 proteins, we used the GAL4 yeast two-hybrid (Y2H) assays with the yeast (*Saccharomyces cerevisiae*) strain AH109 for examination. Growth on SD/–Ade/–His/–Leu/–Trp/X-α-Gal medium plates and blue colony color confirmed that LIF1 indeed interacted with SlWRKY16 (Figure 6C). To verify the interaction between LIF1 and SlWRKY16 proteins and their subcellular localizations when they interact with each other, we also co-expressed the fusion proteins LIF1-GFP and SlWRKY16-RFP. We found that GFP and RFP signals are located mainly in the nucleus when we only overexpress LIF1-GFP or SlWRKY16-RFP in separate *N. benthamiana* leaves (Figure 6D). However, when we co-express LIF1-GFP and SlWRKY16-RFP in the same leaves, GFP and RFP signals mostly overlap in the cytoplasm (Figure 6D). These results not only further confirm that LIF1 and SlWRKY16 proteins may interact with each other and become cytosol localized, but also validate our hypothesis that SlWRKY16 can regulate LIF1 expression at the protein interaction level.

### Analysis of the *Cuscuta* signal using *Cuscuta* extract injections

To further discern the nature of the major signals that trigger lignification-based resistance, we injected the first internode of the resistant H9553 with *Cuscuta* extracts subjected to different treatments (Supplemental Figure S15). *Cuscuta* *campestris* extracts were isolated from the stem tissue of *C. campestris* growing on H1706 tomato plants. Untreated or filtered *Cuscuta* extract injections induced the accumulation of lignin in the cortex region (Supplemental Figure S15, B and C). On the other hand, alteration of *Cuscuta* extract pH from 5.8 to 9 abolished lignin accumulation (Supplemental Figure S15, D and E), suggesting either instability or sequestration of the *Cuscuta* signaling molecules in alkaline conditions. In addition, heat-treated extract and protease-treated extract could not trigger the lignification response (Supplemental Figure S15, F–J). Moreover, *Cuscuta* extract injections also induced lignin accumulation in H1706 with VGE overexpressing *CuRLR1*, but not in H1706 with GUS VGE (Supplemental Figure S16). This result indicates that *CuRLR1* may be able to either sense some unknown factors in *Cuscuta* extract or some part of the response to these factors leading to lignin-based resistant responses. Furthermore, filtration of extracts through devices with different molecular weight cutoffs indicates that fractions smaller than 30 kDa cannot trigger strong lignification response (Supplemental Figure S17). Thus, the active *Cuscuta* signal for induction of lignin-based resistance is larger than 30 kDa but smaller than 100 kDa, and distinct from the previously identified *Cuscuta* signal that binds *CuRe1* (Hegenauer et al., 2016, 2020).

## Discussion

*Cuscuta* spp. cause massive losses in infested tomato fields in the USA, so understanding the resistance mechanism of these specific Heinz tomatoes will provide the potential of developing crop protection systems. Notably, previous studies indicate that different *Cuscuta* species can have diverse host–parasite interactions with the same host species (Ranjan et al., 2014; Kaiser et al., 2015; Hegenauer et al., 2016). For example, although cultivated tomatoes (*S. lycopersicum*) are generally resistant to *Cuscuta reflexa* (Sahm et al., 1995; Hegenauer et al., 2016), most domesticated tomato cultivars are susceptible to *C. campestris*. Therefore, using the Heinz tomato cultivars that have been bred for resistance to dodders helped us understand the multilayered resistance mechanisms to *Cuscuta* spp. and how this might aid in developing parasitic plant-resistant crops. This study reveals the underlying resistance mechanism is a lignin-based resistance response in these Heinz resistant tomato cultivars.

Lignin is a complex phenolic polymer, which is generated from three major monolignols, paracoumaryl alcohol, coniferyl alcohol, and sinapyl alcohol, using covalent crosslinks formed via free radical polymerization (Ferrer et al., 2008). Accumulation of lignin in plant stems or roots has been shown to reinforce plant resistance to invading herbivores, parasites, and pathogens (Reimers and Leach, 1991; Gayoso et al., 2010; Taheri and Tarighi, 2012; War et al., 2012; Dhakshinamoorthy et al., 2014; Kumari et al., 2016; Zhang et al., 2019). Ligniﬁcation at the host–parasite interface in roots has been reported in plants that are resistant to root parasitic plants (Goldwasser et al., 1999; Pérez-de-Luque et al., 2005; Cameron et al., 2006; Lozano-Baena et al., 2007). However, for stem parasitic plants, most research has focused on hypersensitive response or necrosis as the major mechanisms for host plant defense (Lane et al., 1993; Hegenauer et al., 2016; Su et al., 2020). One previous report of incompatible reactions between tomato plants and *C.* *reflexa* characterized by a visible brownish plaque at infection sites suggested this might be due to suberized or lignified cell walls (Sahm et al., 1995). Here, we first identified a strong lignin-based resistance response toward *C. campestris* attack in these specific Heinz tomato cultivars, adding another layer on the previous reported hypersensitive-type response mechanism.

This lignin-based resistance response is regulated by three key genes, *LIF1*, *SlMYB55*, and *CuRLR1*. Of these, *CuRLR1* responded to unknown *Cuscuta* signals and further reinforced lignin deposition in the resistant cultivars. The *Cuscuta* signals that trigger the lignin-based defense responses appear to be large heat-sensitive proteins (30–100 kDa, Supplemental Figure S17), and distinct from the previously identified small *Cuscuta* signal 11 kDa glycine-rich protein or its minimal peptide epitope Crip21 (Hegenauer et al., 2020) that is recognized by *CuRe1* (Hegenauer et al., 2016). It would be of interest to investigate interactions between these potential *Cuscuta* signals or effectors that interact with the two different *Cuscuta* receptors.

**Figure 7:**
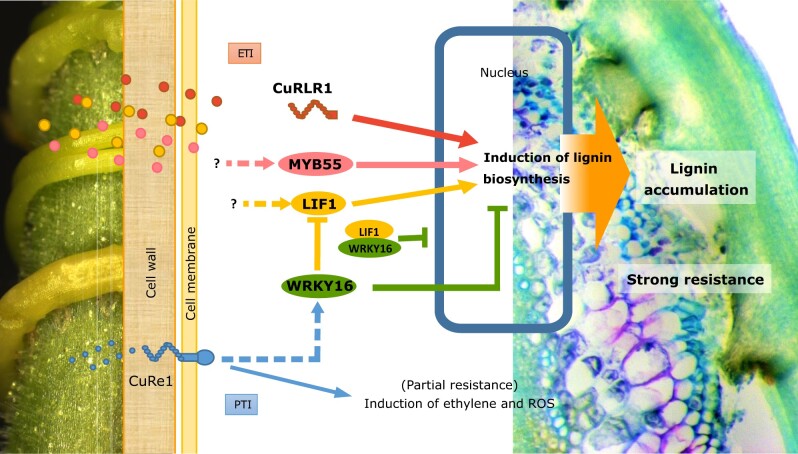
Model of *C. campestris* resistance response in tomato cultivars. Red-labeled pathway: identified cytosolic *CuRLR1*, which may receive large signaling molecules from *C. campestris* or play a role in signal transduction upon *Cuscuta* perception. This triggers downstream signal transduction and induces a lignin-based resistance response. This resistant response may be an ETI. Pink- and yellow-labeled pathway: *SlMYB55* and *LIF1* function as positive regulators in the lignin biosynthesis pathway. Yellow- and green-labeled pathway: *SlWRKY16-* and *LIF1-*mediated lignin-based resistant responses and with a potential connection to *CuRe1*. Blue-labeled pathway: previously identified *CuRe1-*mediated PAMP/MAMP-triggered immunity pathway. ROS, reactive oxygen species.

In conclusion, we propose a multilayered model for *Cuscuta* resistance response in tomato (Figure 7). *CuRLR1* is a cytosolic factor, which either receives large signaling molecules from *C. campestris* as a intracellular receptor or may be a previously undescribed factor which plays a role in downstream signal transduction upon sensing *Cuscuta* signals, and triggers a lignin-based resistance response (Figure 7, red-labeled arrow). Based on previous studies, NBS-LRRs are usually located in the cytoplasm and nucleus and likely to recognize pathogen effectors to induce effector-triggered immunity (ETI; Dodds and Rathjen, 2010). This matches where we observed CuRLR1 subcellular localization (Figure 6) and might also explain why the *Cuscuta* signals that trigger the lignin-based defense responses are in a different size range compared with the previously identified *Cuscuta* signal. Our research results shed light on a potential ETI pathway in parasitic plant resistance and provides the foundation for future studies into how these various layers of resistance connect.

In our model, *SlMYB55* and *LIF1* were placed as positive regulators in the lignin biosynthesis pathway (Figure 7, pink and yellow-labeled arrows) because transient overexpression of *SlMYB55* and *LIF1*-induced lignin accumulation in the cortex (Figure 3). Other yet undiscovered *Cuscuta* receptors or factors may induce *SlMYB55* and *LIF1* expression upon *Cuscuta* attachment. On the other hand, *wrky16* plants showed lignin accumulation and stronger resistance to *Cuscuta*, suggesting that *SlWRKY16* is a negative regulator of this lignin-based resistance pathway (Figure 7, green-labeled arrow). Based on our DNA-Seq, BiFC, and subcellular localization data (Figure 6;  Supplemental Figure S9), we propose that *SlWRKY16* might regulate the function of *LIF1* by physical capture of LIF1 proteins to block their entry into the nucleus (Figure 7, yellow and green-labeled arrows). Furthermore, whether the resistance-specific SNPs in the *LIF1* promoter region influence WRKY or other TFs binding affinity would be of interest for future research and could reveal transcriptional regulatory mechanisms that might influence *LIF1* expression levels. *CuRe1* is reported to mediate PAMP/MAMP-triggered immunity (Hegenauer et al., 2016; Figure 7, solid blue-labeled arrow). GCN analysis indicates a coexpression connection between *CuRe1* and *SlWRKY16* (Figure 4, A–D). *CuRe1* and *SlWRKY16* both became central hub genes in resistant cultivars upon *Cuscuta* attachments (Figure 4D), suggesting the hypothesis that *SlWRKY16* may act downstream of *CuRe1* (Figure 7, dashed blue-labeled arrow). Thus, we envision crosstalk between different resistance pathways that may be triggered together to enhance host defense responses.

We conclude from our work that the resistance in these specific Heinz tomato cultivars relies on a lignin-based response. The systematic investigation of this resistance response in tomato plants toward the stem parasitic plant *C. campestris* provides potential implications for enhancing crop resistance to parasitic plants. Interestingly, none of the early-step lignin biosynthetic genes, like the genes encode phenylalanine ammonia lyase (PAL), cinnamate 4-hydroxylase (C4H), 4-coumarate CoA ligase, were in the model-based DEG list. Changing the early steps in lignin biosynthetic genes can also change the phenylpropanoid pathway for the biosynthesis of anthocyanins. This further confirms that lignin biosynthesis is specifically triggered in the Heinz-resistant cultivars. Notably, the overexpression of the CuRLR1 protein-induced upregulation of lignin precursors, but extensive lignin accumulation was only triggered by *Cuscuta* extracts. Introducing CuRLR1 protein could provide resistance to *C. campestris* without triggering the crop to continuously spend a lot of resources producing a large amount of cortical lignin with associated stunted growth. The identification of *CuRLR1* might provide a path forward to introduce resistance into other important crops that are also attacked by *C. campestris*. In summary, *CuRLR1*, *SlWRKY16*, *LIF1*, and *SlMYB55* regulate a lignin-based response in the tomato stem cortex, which prevents *C. campestris* strands from parasitizing these resistant Heinz cultivars.

## Materials and methods

### Plant materials used in the study

We obtained four different tomato (*Solanum lycopersicum*) cultivars from HeinzSeed, including the resistant H9492 and H9553, and the related susceptible cultivar H9775 and the sequenced susceptible cultivar H1706. Our *Cuscuta* was originally collected from tomato field in California, and we obtained seeds from W. Thomas Lanini. This *Cuscuta* was previously identified as *Cuscuta pentagona* (Goldwasser et al., 2001), which is a closely related species to *C.* *campestris* (Costea et al., 2015). To clear up the confusion, we extract DNA to verify species by molecular phylogenetics. Based on phylogenetic analysis of plastid trnL-F, rbcL sequences, and nrITS, nrLSU sequences (Stefanović et al., 2007; García et al., 2014; Costea et al., 2015), we confirmed that our experimental species is *C.* *campestris* (Supplemental Figures S18–S22). According to our results, our *C.* *campestris* isolate is most similar to *C.* *campestris* 201 voucher Rose 46281 WTU from USA, CA (Supplemental Figure S22) that is published (Costea et al., 2015).

### Histology and cell wall-specific staining

For preparing the sections at the *C. campestris* attachment area and Agroinjection sites on tomato stems, we hand-sectioned plants at 200- to 500-μm thickness using razor blades and kept these sections in 4°C water before staining. For preparing the sections of haustoria attached to host, we fixed samples in 7% w/v Plant Tissue Culture Agar and used Lancer Vibratome Series 1000 to prepare 100-μm sections and kept these sections in 4°C water.

For Ph–HCl staining, we followed the published protocols (Liljegren, 2010; Pradhan Mitra and Loqué, 2014) with some modifications. To prevent plasmolysis during staining, we added an ethanol dehydration process before staining, which is described as follows: we removed the water and then immersed sections in cold 30% ethanol and then 60% ethanol for 5 min each. We prepared Ph–HCl stain or Wiesner stain by preparing a 2:1 mixture of 100% EtOH and concentrated HCl and dissolving powdered phloroglucinol into this solution at a final concentration of 3% w/v. After removing the 60% ethanol, we added Ph–HCl solution dropwise to the Petri dishes, and let the sections sit in the stain for 5 min. The lignified areas of the sections stain bright red within 30 s of immersion in the stain. After removing the Ph–HCl and adding 60% ethanol back, we imaged the sections in the petri dish on a white background using a Zeiss SteREO Discovery, V12 microscope and Nikon Eclipse E600 microscope.

For Toluidine Blue O staining, we used a published protocol with some modifications (O'Brien et al., 1964). We immersed the sections in the stain for 30 s, and then washed with water three times for 30 s each. After removing the agar from around the sections using forceps, we mounted the sections with water on a slide and imaged using a Nikon Eclipse E600 microscope.

### Image analysis of stem and haustorium sections

To quantify the lignin content of each section, we analyzed images using the image processing software ImageJ (Schneider et al., 2012). We added a Gaussian blur with a sigma radius of 2.00 to reduce image noise. We set the color space of the image to L*a*b* to generate histograms that measure lightness, green–red contrast, and blue–yellow contrast of the image. We adjusted the lightness filter to allow histogram coordinates ranging from zero to the peak of the image histogram, and the green–red filter to allow from the histogram peak to 255, and the blue–yellow filter to allow all histogram coordinates. These coordinates filter for red areas on the image, corresponding to lignified areas in the stem sections. We measured the total area of lignification, then selected areas corresponding to the lignified xylem of the stem, and measured this area. We subtracted the xylem area from the total lignin area to calculate the cortex lignin area.

### DNA-seq library construction for resistant and susceptible Heinz cultivars

DNA was extracted from the leaves of three weeks old seedlings using GeneJET Plant Genomic DNA Purification Mini Kit (Thermo Scientific, Waltham, MA, USA) DNA-Seq libraries were prepared using an in-house protocol modified from Breath Adapter Directional sequencing (BrAD-seq; Townsley et al., 2015). First, 5 µg of genomic DNA was fragmented using a Covaris E220 (Covaris, Inc. Woburn, MA, USA) with the following settings: Peak Incident Power (W) 140; Duty Factor 10%; Cycles per Burst 200 and Treatment Time (s) 90 to obtain an average fragment size of 400 base pairs. Next, the fragmented DNA was end-repaired and A-tailed in a single reaction using DNA End Repair Mix and Taq DNA polymerase (New England Biolabs). Y-type adapters were ligated and an enrichment PCR was performed with as in BrAD-seq (Townsley et al., 2015) using seven cycles. Individual libraries were quantified by PCR and pooled to equaled amounts. After a final library cleanup with AMPure beads (Beckman Coulter, Brea, CA, USA), DNA-seq libraries were sequenced at the California Institute for Quantitative Biosciences (QB3) at the University of California, Berkeley using the HiSeq 4000 platform at 150 paired read (Illumina Inc. San Diego, CA, USA).

### Resistant and susceptible Heinz cultivar DNA-seq SNP analysis, promoter binding site analysis, and protein domain and structure prediction

For SNP analysis, we mapped DNA-seq read data to sequenced H1706 tomato genome itag 3.0 to identify SNPs using CLC Genomics Workbench 11 (QIAGEN, https://www.qiagenbioinformatics.com/). Next, we compared the SNPs across the resistant and susceptible tomato cultivars and focus on finding the SNPs that are common in H9553 and H9492 but different from H9775. In other words, we focus on the SNPs that exist in resistant cultivars and named these SNPs as “resistant specific SNPs” (Supplemental Data Set S5). Among our four candidate genes, *LIF1* was the only gene that has resistant-specific SNPs in the promoter region (Supplemental Data Set S6). In order to identify potential introgression regions, we conducted phylogenetic network analysis using the PhyloNetworks package (Solís-Lemus et al., 2017) in the Julia environment on Extreme Science and Engineering Discovery Environment (XSEDE) (Dahan et al., 2014) with 500 kb of sequence around the LIF1 resistance-specific SNP-enriched region (Supplemental Figure S8). To identify potential TF binding sites in the *LIF1* promoter region, we used PlantPAN 3.0 (http://PlantPAN.itps.ncku.edu.tw; Chow et al., 2015; Chow et al., 2018) “TF/TFBS Search” and “Promoter Analysis”. We also predicted the *SlWRKY16* binding site based on the homologous genes in the phylogenetic tree of WRKY domains on the Plant Transcription Factor Database (PlantTFDB v5.0, http://planttfdb.gao-lab.org; Phylogenetic Tree for Solanum lycopersicum WRKY Family: http://planttfdb.gao-lab.org/phylo_tree.php?sp=Sly&fam=WRKY;  Jin et al., 2016). To determine the consequences of the K251Q amino acid replacement in the LIF1 protein, we predicted potential protein domains of LIF1 using InterProScan (https://www.ebi.ac.uk/interpro/search/sequence/;  Jones et al., 2014; Mitchell et al., 2018). We conducted protein 3D structure prediction and analysis using Phyre2 (Protein Homology/analogY Recognition Engine v 2.0, http://www.sbg.bio.ic.ac.uk/phyre2/html/page.cgi?id=index;  Kelley et al., 2015). We also predicted whether K251Q amino acid substitution is deleterious or neutral using PROVEAN (Choi, 2012; Choi et al., 2012; Protein Variation Effect Analyzer, http://provean.jcvi.org/seq_submit.php).

### Time-course RNA-seq library construction and analysis

We challenged H1706 tomato cultivars with strands of *C. campestris* and collected stem tissues at 1, 2, 3, 4 DPA and 0 DPA as negative controls. Following this, we constructed strand-specific poly-A based libraries for RNA-seq (Townsley et al., 2015). We conducted sequencing of these libraries on two lanes on Illumina HiSeq 2000 at 50-bp single read (SR).

We used using CLC Genomics Workbench 11 (QIAGEN) for following RNA-seq analysis. First, we mapped resistant and susceptible cultivar RNA-seq read data to sequenced H1706 tomato genome itag 3.0. To see the general pattern across libraries, we conducted PCA of gene expression across different DPA. Next, we used ANOVA comparison with DPA factors and cutoff FDR <0.1 to select DEG list. Then, we drew Venn diagrams of DEGs at different DPA libraries. 0 DPA libraries are without *Cuscuta* treatments and serve as the negative control for comparisons. The cutoff of these DEGs is FDR < 0.1 and fold change > 1.5. Following, we constructed a heat map of DEGs across different DPA libraries. Euclidean distance and complete linkage are used for this clustering analysis (Supplemental Figure S1).

### Resistant and susceptible cultivar RNA-seq library construction and interaction model-based analysis

We challenged the resistant and susceptible tomato cultivars with strands of *C. campestris* and collected stem tissues at 4 DPA. Following this, we constructed strand-specific poly-A-based libraries for RNA-seq from the four tomato cultivars, including the resistant cultivars H9492 and H9553, and the susceptible cultivars H9775 and H1706. We conducted sequencing of these libraries on two lanes on Illumina HiSeq 4000 at 100-bp SR. We first mapped reads to sequenced H1706 tomato genome itag 2.4. To investigate gene expression changes across the resistant and susceptible cultivars in dodder infested versus uninfested plants, we conducted a PCA with K-means clustering using the normalized read counts of sequences mapped to the tomato transcriptome (Supplemental Figure S2). Next, we defined DEGs with the Bioconductor package DESeq2 employing an interaction design (design = ∼ Condition + Genotype + Condition: Genotype). Following this, we focused on these 113 genes that display expression changes upon dodder infestation that are different in H9492 and H9553 compared to H1706 and H9775. Within these 113 genes, we picked our three candidate genes based on gene annotation and functions.

### Barnes–Hut clustering analysis and GCN analysis for resistant and susceptible cultivar RNA-Seq

In order to get a more comprehensive DEG list, we mapped resistant and susceptible cultivar RNA-seq read data to sequenced H1706 tomato genome itag 3.0 by using CLC Genomics Workbench 11 (QIAGEN). Next, we used ANOVA comparison with both factors, all cultivars and with/without *Cuscuta* treatments, and cutoff FDR <0.1 to select DEG list. In these 10939 genes, we applied BH t-SNE using RSMod package (script included in code availability) generated 85 gene clusters based on their gene expression patterns. Based on their GO enrichment terms and their included candidate genes, five clusters were selected for further analysis (Supplemental Data Set S9, yellow-labeled genes). We use these selected genes to build GCNs by using the R script (script included in code availability) that was modified from our previously published method (Ichihashi et al., 2014). We constructed GCNs for different *C. campestris* treatments in susceptible and resistant cultivars with normal quantile cutoff = 0.997.

### VGE and VIGS in tomatoes

For preparing the binary vector for plant transient expression that carries the ToMoV DNA, we used a modified pSP72-TAV (Gilbertson et al., 1993; Hou and Gilbertson, 1996) that is lacking the capsid protein (CP) ORF and has a restriction enzyme multisite in which a Gateway cassette (Thermo Fischer Scientific) was cloned by In-Fusion (Takara) in the NcoI site. The whole replicon fragment of TAV-GW was amplified from this vector and cloned into a binary vector to generate a vector for *Agrobacterium*-mediated transient expression in plants (pMR315). Since the ToMoV DNA-B (carrying the viral movement protein) and the CP are missing, this clone is not an infectious clone and it only serves as a viral replicon by replicating via rolling circle mechanism (Stenger et al., 1991). The gene cloned into this vector is driven by the CP promoter, which is in the nontranslated region between the end of the common region and the start codon of the CP gene that was removed (Supplemental Figure S3).

For transiently overexpressing our candidate genes in the susceptible tomato cultivar H1706, we used this VGE vector pTAV (Supplemental Figure S3). We cloned *GUS*, *LIF1*, *SlMYB55*, and *CuRLR1* genes into pTAV and transformed these into thermo-competent *Agrobacterium tumefaciens* by heat-shock transformation. For culturing *Agrobacterium* and preparing agroinjection, we followed the previously published protocol (Velásquez et al., 2009) with some modifications. For each experiment, we started from growing transformed *Agrobacterium* on lysogeny broth (LB) agar plates with appropriate antibiotic selections at 30°C for 2 d. Following this, we inoculated 10-mL liquid LB with transformed *A.* *tumefaciens* (AGL1) and incubated at 30°C for 16 h with 200 rpm. shaking. We diluted the primary cultures 1:5 into Induction Media (Velásquez et al., 2009) supplemented with appropriate antibiotic selections and 200-µM acetosyringone, and then incubated them at 30°C for 24 h with 200 rpm. shaking. When the optical density (OD_600_) of the culture was around 1, we harvested the transformed *A. tumefaciens* (AGL1) by centrifuging at 5000 rpm for 10 min and then resuspended agrobacteria in inoculation buffer (10-mM 2-[N-Morpholino] ethane sulfonic acid (MES), 10-mM MgCl_2_, 200-µM acetosyringone, and 0.5-mM dithiothreitol) to an OD_600_ of 1 culture. Next, we injected this transformed *Agrobacterium* culture into the first internode of tomato stems using a syringe equipped with a 0.8 mm × 38.1 mm MonoJect needle.

For VIGS, we followed the published VIGS in tomato protocol and tobacco rattle virus (TRV)-based vector system (Liu et al., 2002) with slight modifications. This TRV-based VIGS contains TRV-RNA1 (pTRV1) and TRV-RNA2 (pTRV2), which includes multiple cloning sites for building constructs for the genes of interest. Transformed pTRV1 and pTRV2 *Agrobacterium* cultures were mixed in a 1: 1 ratio before infiltration. After about 3.5 h of incubation on the shaker at room temperature, mixed *Agrobacterium* cultures were infiltrated onto the cotyledons of 9-d-old tomato plants using a 1-mL needleless syringe.

### Preparation of *C. campestris* extracts and injection protocols

For one mL *C. campestris* extracts, we collected 100 mg of the stem tissue in microcentrifuge tubes from *C. campestris* growing on H1706 tomato plants. We used the BioSpec Mini-Beadbeater to grind the liquid nitrogen-frozen tissue with five 2.3-mm diameter BioSpec zirconia beads and 1.0-mm diameter BioSpec zirconia beads in the tubes for 1 min, and then mixed with 1-mL deionized water. To remove the plant tissue debris, we centrifuged extracts for 30 s at 5,000 r.c.f., and used only the supernatant for untreated extract injections. For heat-treated extracts, we heated at 95°C for 5 min. For pH treated extracts, we adjusted the pH to 9 by adding 0.1-M NaOH. For filtered extracts, we filtered untreated extracts through a VWR 0.2-µm sterile syringe filter. We injected different treated extracts into the first internode of tomato stems using a syringe equipped with a 0.8 mm × 38.1 mm MonoJect needle. Furthermore, we used 3K, 10K, 30K, and 100K Amicon Ultra Centrifugal Filter Devices to filter *Cuscuta* extracts. Then, we use flow through extracts to do injection on H9553 stems to test the size of *Cuscuta* signals.

### Protein interaction, subcellular localization, and co-localization of fusion candidate proteins

For protein interaction assays, we performed in vivo using BiFC system (Kerppola, 2008). The plasmids were constructed by using the Gateway-compatible BiFC vectors SPDK1794 (*p35S::cCitrine*) and SPDK1823 (*p35S::nCitrine*). The leaves of 4-week-old *N.* *benthamiana* were injected with different combinations of *A.* *tumefaciens* GV3101 containing the transient expression vectors (Fang and Spector, 2010).

For subcellular localization and co-localization of fusion candidate proteins, we performed with transient expression fluorescent fusion proteins in vivo. The plasmids were constructed by using the Gateway-compatible vectors pGWB5 (*p35S::GFP*) and pGWB660 (*p35S::TagRFP*). The leaves of 4-week-old *N. benthamiana* were injected with the *A. tumefaciens* GV3101 strain containing one of the plasmids (Sparkes et al., 2006). To verify the interaction between LIF1 and SlWRKY16 proteins and determine their subcellular localizations when they interact with each other, we also co-expressed the fusion proteins LIF1-GFP (*p35S::LIF1-GFP*) and SlWRKY16-RFP (*p35S::SlWRKY16-TagRFP*). To reduce gene‐silencing and enhance transient expression of our candidate proteins, we co-expressed the fusion proteins with p19 obtained from Professor Bo Liu’s Lab at University of California, Davis. Three individual plants and three adult leaves of each plant were used for each treatment. Fluorescence was observed 2–5 d after transfection by a Confocal Laser Scanning Platform Zeiss LSM710 (Zeiss, Germany) with following settings: excitation laser wavelength 488 nm (for GFP) and 561 nm (for RFP), intensity 40%, detection wavelength 493–530 nm (for GFP), and 578–645 nm (for RFP), detector gain: 700.

### Validation of protein–protein interaction by yeast two-hybrid analysis

To validate the predicted protein–protein interaction between two of our candidate TFs, AP2 and WRKY16, we used the GAL4 yeast two-hybrid system (Clontech). AP2 and WRKY16 were cloned into pGADT7-GW (Addgene Plasmid #61702) and pGBKT7-GW (Addgene Plasmid #61703) plasmids (Lu et al., 2010). Empty pGADT7 (with the GAL4 activation domain, AD) and pGBKT7 (with the GAL4 DNA-binding domain, BD) plasmids were used as negative controls. We use the yeast (*Saccharomyces cerevisiae*) strain AH109 in the MATCHMAKER GAL4 Two-Hybrid System (Clontech), in which *HIS3*, *ADE2*, *MEL1*, and *LacZ* are under the control of GAL4 TF. The AD and BD plasmids were co-transformed to yeast AH109 competent cells following Clontech’s user manual instructions of the polyethylene glycol/lithium acetate method and cultured in YPD Plus Medium for 90 min to promote transformation efficiency. Transformed yeast cells were assayed by culturing in SD/–Leu/–Trp medium to select for successful co-transformants and then assayed by culturing in SD/–Ade/–His/–Leu/–Trp medium with 40 μg·mL^−1^ X-α-Gal, which provides high-stringency selection. The positive protein–protein interactions between two TFs are indicated by growth on SD/–Ade/–His/–Leu/–Trp/X-α-Gal medium plates and blue colony color.

### Lignin content and composition analysis

Tomato stem material from specified treatments was flash-frozen in liquid nitrogen and lyophilized at −50°C and ≤0.1 mbar for 48 h in a 6L FreeZone 6 Benchtop Freeze Dry System (Labconco Corp., Kansas City, MO, USA). Lyophilized material was pulverized in a 2-mL screw top microcentrifuge tube with a glass bead for 10 min at a frequency of 20 s^−1^ in a TissueLyser (Retsch ballmill, Qiagen, Venlo, Netherlands). The material was AIR prepped and destarched using protocols from Barnes and Anderson (2017). Acetyl bromide analysis was carried out using the same protocol with one exception: Samples were incubated at 50°C with gentle swirling every 10 min for 2 h to limit the degradation of xylan, which can lead to the over quantification of lignin present in the sample. Lignin quantification reactions were performed in a 10-mm light path quartz cuvette (VWR, Radnor, PA, USA, catalog number 414004-062) and absorbance measurements were taken on a SPECTRAmax plus 384 (Molecular Devices, San Jose, CA, USA). Acetyl bromide soluble lignin (%ABSL), which indicates percent absorbance of soluble lignin, was calculated using the extinction coefficient of 17.2 (Chen and Dixon, 2007). Presently, no extinction coefficient is available for tomato stem lignin so the extinction coefficient for *N.* *benthamiana* stem lignin was used.

Cell wall-bound aromatics and HPLC analysis of liberated aromatics was performed as described by Eudes et al. (2015). The remaining cell wall material post-aromatic extraction, now enriched for lignin, was then washed with water three times and dried at 30°C overnight. The chemical composition of the lignin-enriched cell wall material was analyzed by pyro-GC-MS using a previously described method with some modifications (Eudes et al., 2015). Pyrolysis of biomass was performed with a Pyroprobe 6200 (CDS Analytical Inc., Oxford, PA, USA) connected with GC/MS (GCMS-QP2010 Ultra Gas Chromatograph Mass Spectrometer, Shimadzu corp., Kyoto, Japan) equipped with an AgilentHP-5MS column (length: 30 m, inner diameter: 0.25 mm, film thickness: 0.25 μm). The pyrolysis was carried out at 550°C. The chromatograph was programmed from 50°C (1 min) to 300°C at a rate of 30°C·min^-1^; the final temperature was held for 10 min. Helium was used as the carrier gas at a constant flow rate of 1 mL·min^−1^. The mass spectrometer was operated in scan mode and the ion source was maintained at 300°C. The compounds were identified by comparing their mass spectra with those of the NIST library and those previously reported (Ralph and Hatfield, 1991; Gutiérrez et al., 2006). Peak molar areas were calculated for the lignin degradation products, and the summed areas were normalized per sample.

### Data availability

All data is available in the main text or the supplementary materials. All DNA-Seq and RNA-Seq raw data are deposited on NCBI SRA PRJNA550259.

### Code availability

All R scripts and package for analysis are deposited on GitHub. R script for RNA-Seq interaction model-based analysis deposited on GitHub (Link: https://github.com/MinYaoJhu/Moran-s-RNA-Seq-analysis-script). R script and RSMod package for Barnes–Hut clustering analysis deposited on GitHub (Link: https://github.com/sdrowland/RSMod). R script for RNA-Seq GCN analysis deposited on GitHub (Link: https://github.com/Hokuto-GH/gene-coexpression-network-script).

### Accession numbers

Sequence data from this article can be found in the GenBank/EMBL data libraries under accession numbers NCBI SRA PRJNA550259.

## Supplemental data 

The following materials are available in the online version of this article.

**Supplemental Figure S1.** Analysis of time-course RNA-seq data.

**Supplemental Figure S2.** RNA-seq analysis results of gene expression across resistant and susceptible cultivars at 4 DPA.

**Supplemental Figure S3.** Virus-based gene expression vectors (pTAV) map.

**Supplemental Figure S4.** Expression of candidate genes and VGE of GUS in tomato H1706.

**Supplemental Figure S5.** VIGS in resistant tomato H9553.

**Supplemental Figure S6.** HPLC data for p-coumarate and trans-ferulate levels in different VGE and *C. campestris* infection treatments.

**Supplemental Figure S7.** Haustorium infestation status ratio under different VGE treatments.

**Supplemental Figure S8.** Phylogenetic network analysis using 500-kb sequence around the *LIF1* resistance-specific SNP-enriched region.

**Supplemental Figure S9.** *LIF1* promoter region and TFs binding motifs.

**Supplemental Figure S10.** *SlWRKY16* expression levels under different *Cuscuta* treatment conditions.

**Supplemental Figure S11.** GCN analysis of identified key regulators.

**Supplemental Figure S12.** *PR1*, *DREB1*, and *DREB2* expression levels in *wrky16* and M82.

**Supplemental Figure S13.** *LIF1* expression levels in *wrky16* and VGE overexpressing *SlMYB55* and *LIF1* in H1706, M82, and *wrky16*.

**Supplemental Figure S14.** VGE overexpressing *CuRLR1-*induced stem lignification in susceptible M82 tomatoes but not in *wrky16* tomatoes.

**Supplemental Figure S15.** *Cuscuta campestris* extract injections to detect *Cuscuta* signals.

**Supplemental Figure S16.** VGE overexpressing *CuRLR1* in H1706 with or without *Cuscuta* extract injections.

**Supplemental Figure S17.** *Cuscuta* signal size analysis using *Cuscuta* extract injections.

**Supplemental Figure S18.** Sequence alignment of plastid *trnL-F* intron/spacer region sequences in our *C. campestris* isolate and published *C. campestris* and *C. pentagona*.

**Supplemental Figure S19.** Sequence alignment of plastid ribulose-1,5-bisphosphate carboxylase/oxygenase large subunit (*rbcL*) sequences in our *C. campestris* isolate and published *C. campestris* and *C. pentagona*.

**Supplemental Figure S20.** Sequence alignment of nuclear internal transcribed spacer (*nrITS*) sequences in our *Cuscuta campestris* isolate and published *C. campestris* and *C. pentagona*.

**Supplemental Figure S21.** Sequence alignment of nuclear large-subunit ribosomal DNA (*nrLSU*) sequences in our *Cuscuta campestris* isolate and published *C. campestris* and *C. pentagona*.

**Supplemental Figure S22.** Phylogenetic relationships among our *C. campestris* isolate and published *C. campestris* and *C. pentagona* by maximum-likelihood phylogenies.

**Supplemental Data Set S1.** DEG list of time-course RNA-Seq data.

**Supplemental Data Set S2.** DEG list of resistant and susceptible host response to *C. campestris* by using an interaction design model.

**Supplemental Data Set S3.** Vector pTAV (pMR315_pTAV-GW binary) sequence.

**Supplemental Data Set S4.** Haustorium infestation status quantification.

**Supplemental Data Set S5.** Resistant-specific SNPs in all chromosomes.

**Supplemental Data Set S6.** Resistant-specific SNPs in the *LIF1* promoter region.

**Supplemental Data Set S7.** Predicted TF binding sites in the *LIF1* promoter region.

**Supplemental Data Set S8.** DEG list of four different Heinz tomato cultivar responses to *C. campestris* by ANOVA.

**Supplemental Data Set S9.** Gene list in the BH t-SNE generated clusters.

## Supplementary Material

kiac024_Supplementary_Data

## Notes

M.F. initiated the project with supervision from N.R.S. and established the connection with Heinz. M.-Y.J. conducted phylogenetic analysis and verified *Cuscuta* species. M.-Y.J., M.F., and R.N.P. conducted histochemical and biomass analysis. M.R. modified the VGE vector and M.F. cloned candidate genes into VGE vectors. M.-Y.J., R.N.P., and L.W. conducted VGE overexpression experiments and analysis for all candidate genes. S.D.R. and M.-Y.J. developed lignin section area quantification methods. M.-Y.J. produced VGE overexpressed samples and M.S.B. performed acetyl bromide, high-performance liquid chromatography and Pryo-GC-MS analysis with P.M.S.’s supervision. M.F. made DNA-Seq and RNA-seq libraries. M.F. conducted preliminary SNP analyses and model-based DEG analyses and picked primary candidate genes. M.-Y.J. performed SNP analyses, protein domain and structure prediction, PROVEAN analysis for DNA-Seq data, and ANOVA DEG analyses, BH-SNE clustering to select candidate genes and built GCNs for RNA-seq data. S.D.R. developed the RSMod R package. H.N., S.D.R., and M.-Y.J. modified the GCN analysis script and M.-Y.J. construct GCNs. M.-Y.J. and S.D.R. performed PhyloNetwork analysis. M.-Y.J. and L.W. conducted *wrky16* plant phenotyping and VGE overexpression experiments on *wrky16*. M.-Y.J. and L.W. performed subcellular localization and BiFC experiments. M.-Y.J. performed yeast two-hybrid and protein co-localization experiments. M.-Y.J., R.N.P., and L.W. conducted *Cuscuta* extract injection experiments. M.-Y.J. wrote the initial manuscript draft for the main text and made figures and tables with primary editing from N.R.S. M.-Y.J., R.N.P., L.W., M.S.B., M.R., and H.N. wrote the initial draft for materials and methods. M.-Y.J., M.F., R.N.P., L.W., M.S.B., H.N., K.S.Z., P.M.S., and N.R.S. contributed to the editing of this manuscript. N.R.S. supervised the whole project.

The author responsible for distribution of materials integral to the findings presented in this article in accordance with the policy described in the Instructions for Authors (https://academic.oup.com/plphys/pages/general-instructions) is: Neelima R. Sinha (nrsinha@ucdavis.edu).
