# Supplementary material for: Heinz-resistant tomato cultivars exhibit a lignin-based resistance to field dodder (Cuscuta campestris) parasitism
Source: Plant Physiol. 2022 Jan 31;189(1):129–51. doi: 10.1093/plphys/kiac024 (PMC9070836; doi:10.1093/plphys/kiac024)
Supplement: kiac024_Supplementary_Data [file kiac024_supplementary_data.zip › Supplemental Figures_v5_accepted.pdf]

Supplementary Information for

RESEARCH ARTICLE

Lignin-based resistance to *Cuscuta campestris* parasitism in Heinz resistant tomato cultivars

Min-Yao Jhu[1,2*], Moran Farhi[1,3*], Li Wang[1,4], Richard N. Philbrook[1, 5], Michael S. Belcher[6,7], Hokuto Nakayama[1,8], Kristina S. Zumstein[1], Steven D. Rowland[1], Mily Ron[1], Patrick M. Shih[1,6,9,10], Neelima R. Sinha[1@].

[1] Department of Plant Biology, University of California, Davis, CA, 95616, United States.

[2] Crop Science Centre, Department of Plant Sciences, University of Cambridge, Cambridge, UK.

[3] The Better Meat Co., 2939 Promenade St. West Sacramento, CA, 95691, United States.

[4] College of Life Sciences, Nanjing Normal University, Nanjing, Jiangsu, China.

[5] Dark Heart Nursery, 630 Pena Dr, Davis CA 95616, United States.

[6] Feedstocks Division, Joint BioEnergy Institute, Emeryville, CA, United States.

[7] Department of Plant and Microbial Biology, University of California, Berkeley, Berkeley, CA, United States.

[8] Graduate School of Science, Department of Biological Sciences, University of Tokyo, Hongo Bunkyo-ku, Tokyo, 113-0033, Japan

[9] Genome Center, University of California, Davis, Davis, CA, United States.

[10] Environmental Genomics and Systems Biology Division, Lawrence Berkeley National.

[@] Corresponding author: Neelima R. Sinha

Email: nrsinha@ucdavis.edu.

This PDF file includes:

Supplemental Figure 1 to 21

Legends for Supplemental Data Set 1 to 9

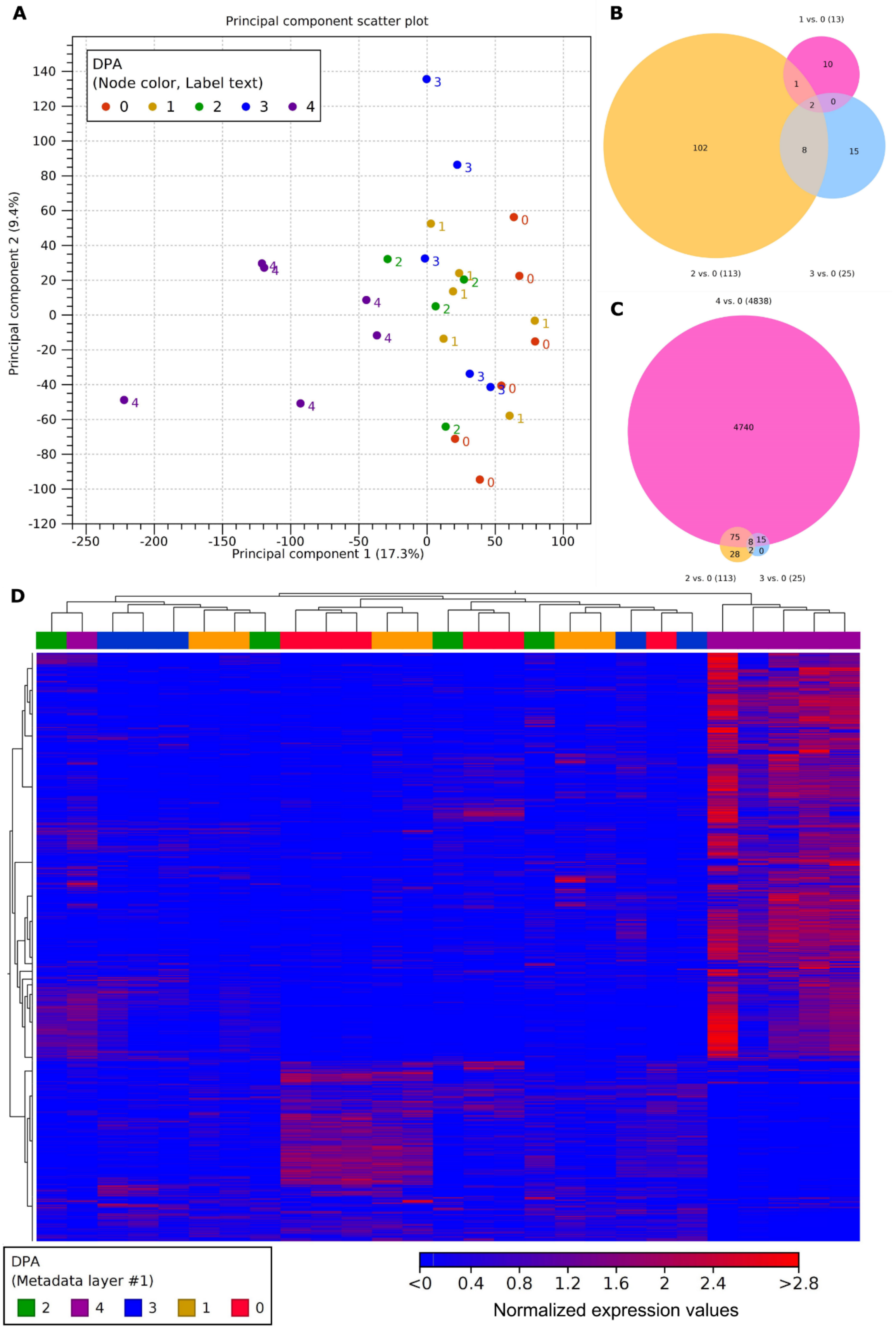
A
Principal component scatter plot
DPA
(Node color, Label text)
0 1 2 3 4
Principal component 2 (9.4%)
Principal component 1 (17.3%)
B
1 vs. 0 (13)
2 vs. 0 (113)
3 vs. 0 (25)
C
4 vs. 0 (4838)
2 vs. 0 (113)
3 vs. 0 (25)
D
DPA
(Metadata layer #1)
2 4 3 1 0
<0 0.4 0.8 1.2 1.6 2 2.4 >2.8
Normalized expression values

Supplemental Figure S1 | Analysis of time-course RNA-Seq data. (A) Principal component analysis (PCA) of gene expression across different day post attachment (DPA). Library number, 0 PDA, n = 6; 1 DPA, n = 6; 2 DPA, n = 4; 3 DPA, n = 5; 4 DPA, n = 6. (B-C) Venn diagram of differentially expressed genes (DEGs) at different DPA libraries. 0 DPA libraries are without *Cuscuta* treatments and serve as the control for comparisons. The cutoff of these DEGs are FDR < 0.1 and fold change > 1.5. (B) Venn diagram of DEGs at 1, 2, 3 DPA libraries. The pink circle indicates the DEGs comparing between 0 DPA and 1 DPA. The yellow circle indicates the DEGs comparing between 0 DPA and 2 DPA. The blue circle indicates the DEGs comparing between 0 DPA and 3 DPA. (C) Venn diagram of DEGs at 2, 3, 4 DPA libraries. The yellow circle indicates the DEGs comparing between 0 DPA and 2 DPA. The blue circle indicates the DEGs comparing between 0 DPA and 3 DPA. The pink circle indicates the DEGs comparing between 0 DPA and 4 DPA. (D) Heat map of DEGs across different DPA libraries. DEGs are selected by ANOVA analysis with cutoff FDR < 0.1. Euclidean distance and complete linkage are used for this clustering analysis.

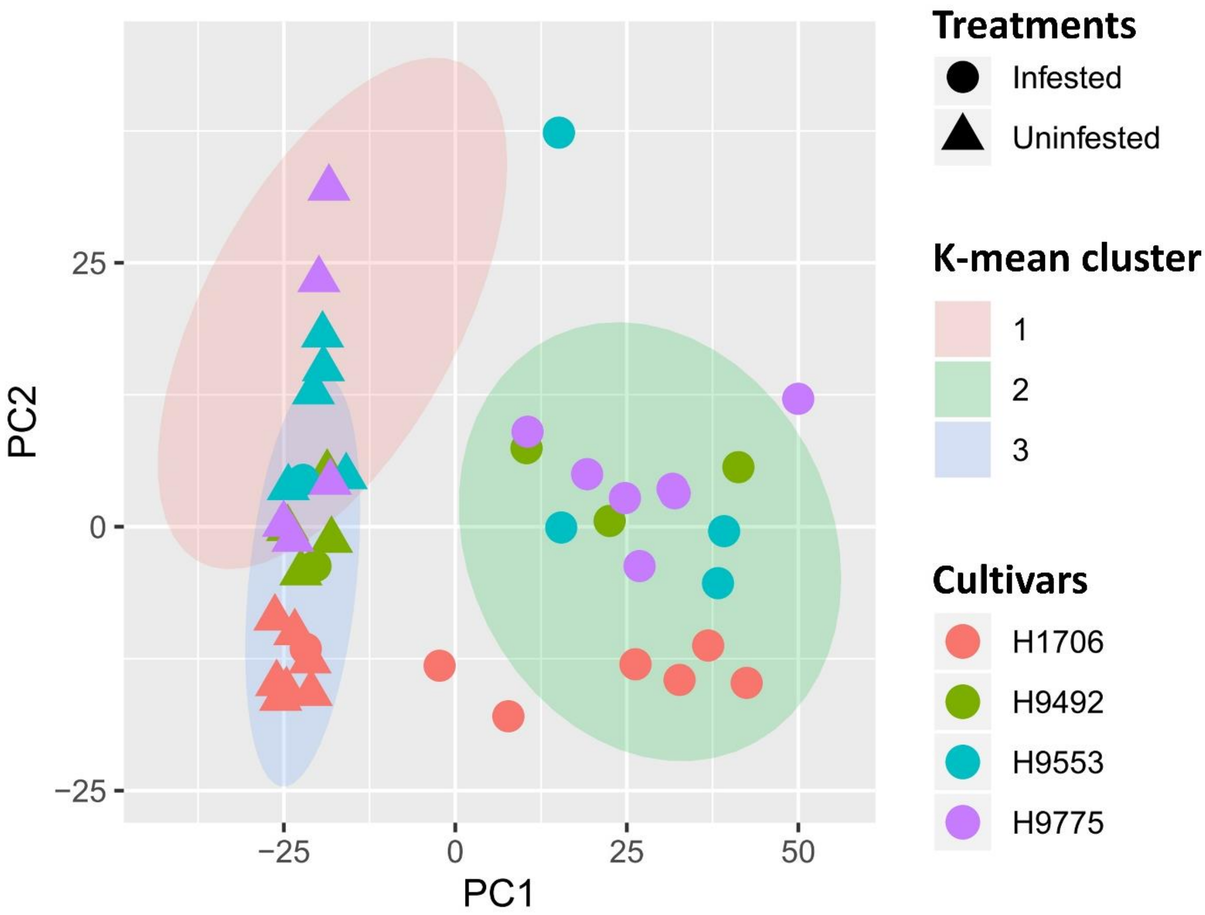

Supplemental Figure S2 | RNA-Seq analysis results of gene expression across resistant and susceptible cultivars at 4 DPA. Principal component analysis (PCA) of gene expression across resistant and susceptible cultivars at 4 DPA. PC1 indicates principal component 1; PC2 indicates principal component 2. Different treatment conditions are represented by shapes: square dots indicate the uninfested host stem tissue samples; circle dots indicate the infested host stem tissue samples. Different cultivars are represented by different colors.

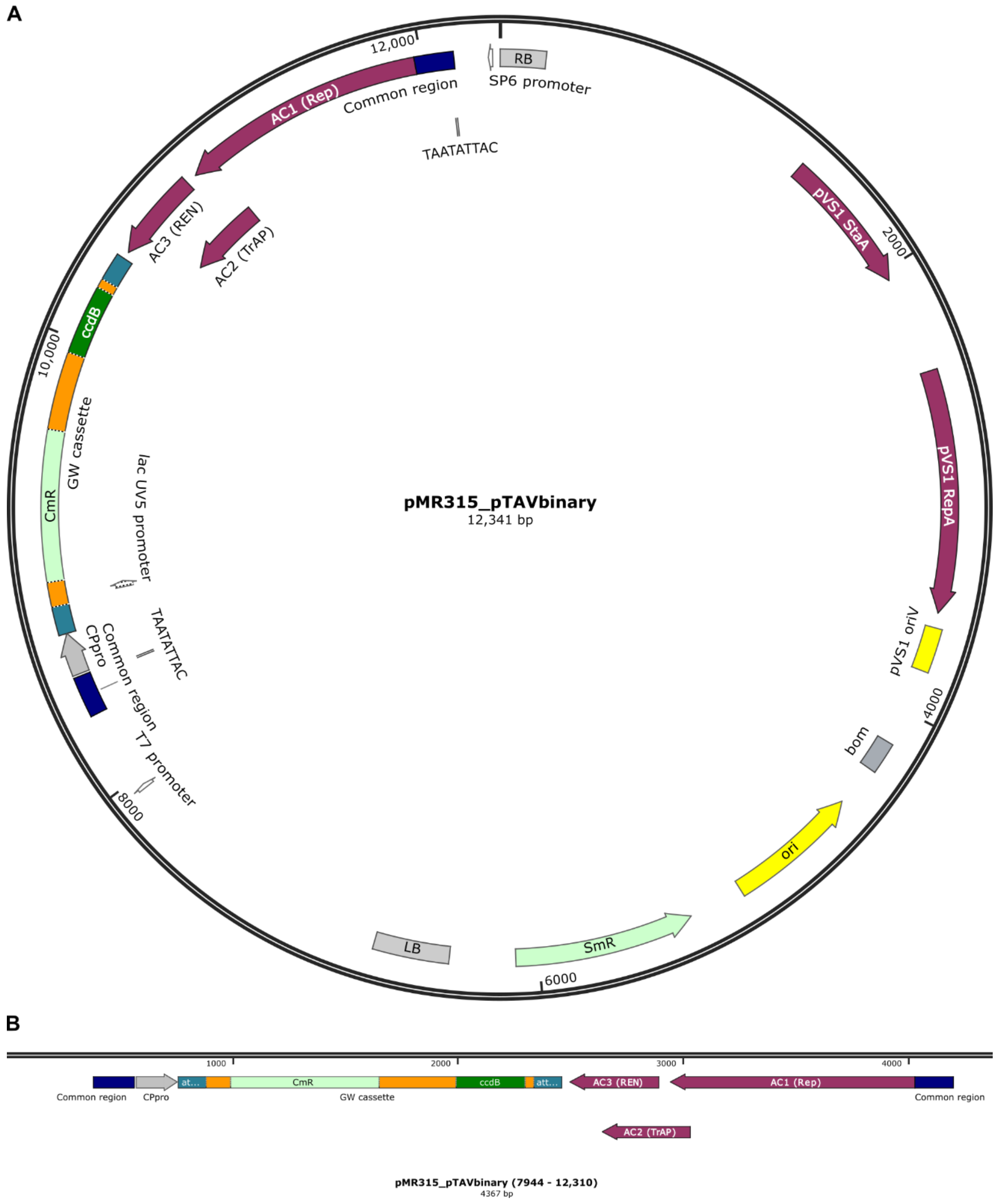

Supplemental Figure S3 | Virus-based Gene Expression vectors (pTAV) map. (A) Full map of pTAV plasmid. (B) Zoom-in view of the Gateway® cassette region. The gene cloned into this vector is driven by the capsid protein promoter (CPpro), which is in the non-translated region between the end of the common region and the start codon of the

capsid protein gene that was removed. CmR indicates chloramphenicol acetyltransferase, which is the Chloramphenicol resistance gene. SmR indicates aminoglycoside adenylyltransferase, which is the Spectinomycin/Streptomycin resistance gene. pVS1 RepA indicates replication protein from the *Pseudomonas* plasmid pVS1. pVS1 StaA indicates stability protein from the *Pseudomonas* plasmid pVS1. Complete sequence of pTAV is attached in Datasets S1.

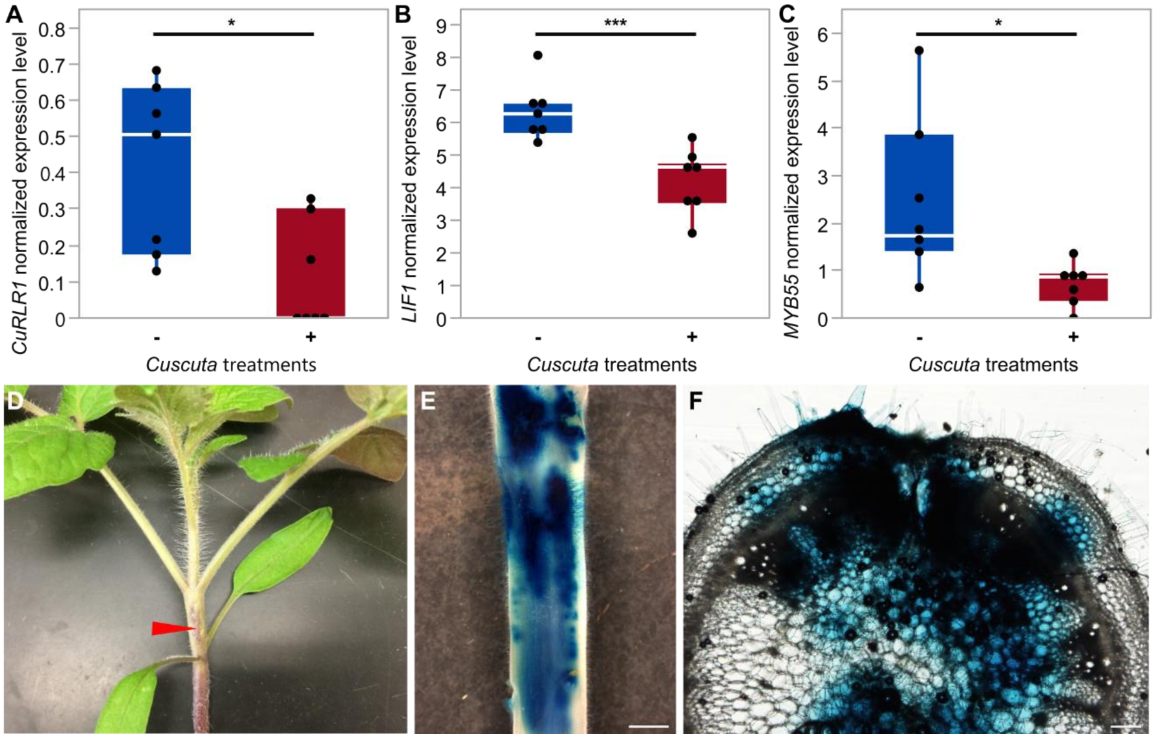

Supplemental Figure S4 | Expression of candidate genes and Virus-based Gene Expression (VGE) of GUS in tomato H1706. (A-C) The normalized expressions levels (CPM, counts per million) of genes in susceptible cultivar H1706 under *C. campestris* infestation. – and + indicates without or with *C. campestris* infection treatments respectively. Biologically independent replicates: RNA-Seq libraries: H1706-Cc, n = 7; H1706+Cc, n = 7. Data are assessed using two-tailed t test. "*": p-values < 0.05, "***": p-values < 0.005. (A-C) The boxplot consists of a box extending from the 25th quantile to the 75th quantile. The centerline in the box indicates the median. The length of the box is the interquartile range (IQR), which is the difference between the 25th quantile and the 75th quantile. The whiskers extend from the ends of the box to the outermost data point that falls within 1.5 times of IQR. Points outside of the whiskers are outliers. (D) Tomato seedling showing first internode. Arrow points to injection site. (E) The first internode of stem stained for GUS expression from VGE construct in susceptible cultivar H1706. Scale

bar, 2 mm. (F) Hand section (about 300 μm) of stem near injection site stained for GUS expression. Scale bar, 200 μm.

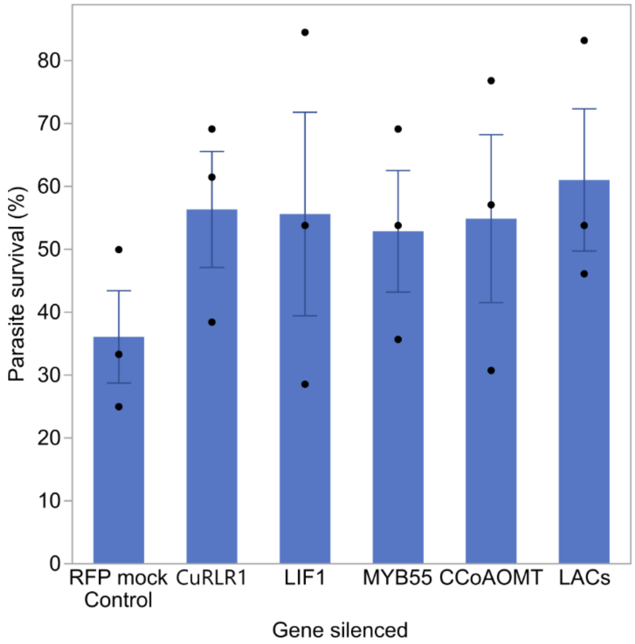

Supplemental Figure S5 | Virus-induced gene silencing (VIGS) in resistant tomato H9553. Plot shows the average *C. campestris* survival rates on different VIGS tomato plants. Survival rates = (the number of *C. campestris* surviving by the end of the experiment/ the total number of *C. campestris* at the beginning of experiment) * 100%. Each dot represents the average survival rate for each experiment, which used 12-14 individual plants (biological replicates) for each VIGSed gene. CCoAOMT, caffeoyl-CoA O-methyltransferase; LAC, laccase. The same VIGS vector with only red fluorescent protein (RFP) construct serves as a mock control for the injection treatment. The error bars indicate standard errors.

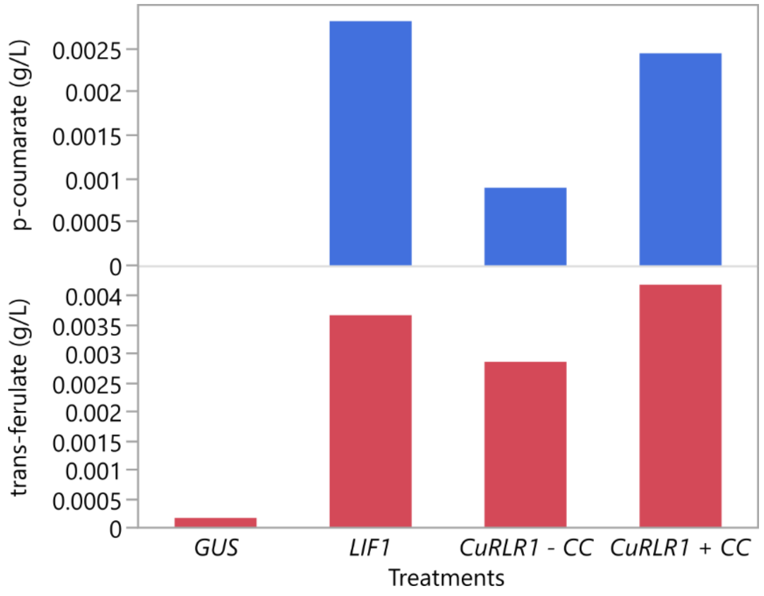

Supplemental Figure S6 | High-performance liquid chromatography (HPLC) data for p-coumarate and trans-ferulate levels in different Virus-based Gene Expression (VGE) and *C. campestris* infection treatments. HPLC data for p-coumarate and trans-ferulate was generated from ethyl acetate extract of de-starched alcohol insoluble residue (AIR) prepped stem tissue. The unit of this data is g/L. –Cc and +Cc indicate without or with *C. campestris* infection treatments respectively. Biological replicates collected from first internodes; *GUS*, n = 8; *LIF1*, n = 8, *CuRLR1*-Cc, n = 18; *CuRLR1*+Cc, n = 18. HPLC assay technical replicate, n = 1.

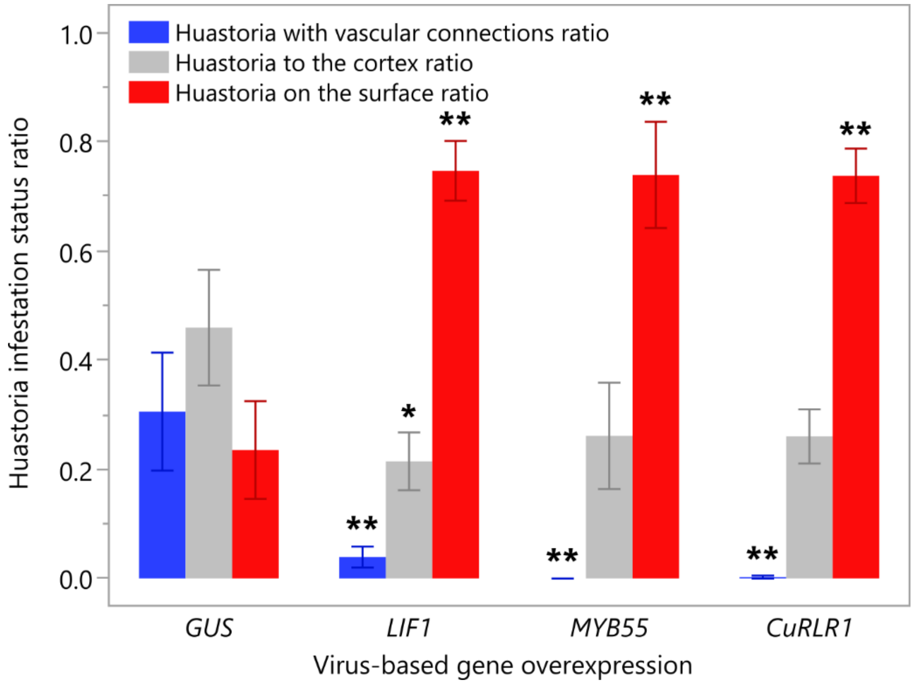

Supplemental Figure S7 | Haustorium infestation status ratio under different VGE treatments. The numbers of haustoria in different infestation status are quantified by examining hand sections and vibratome sections. The ratios are calculated by dividing the number of haustoria in each status by the total number of haustoria on each section. The detailed haustorium number and ratio data are presented in Supplemental Data Set S3. Data are analyzed using Dunnett's test. “*”: p-values < 0.05, “**”: p-values < 0.01. Replicates: *GUS*, n = 18; *LIF1*, n = 35; *MYB55*, n = 11; *CuRLR1*, n = 47. The error bars indicate standard errors.

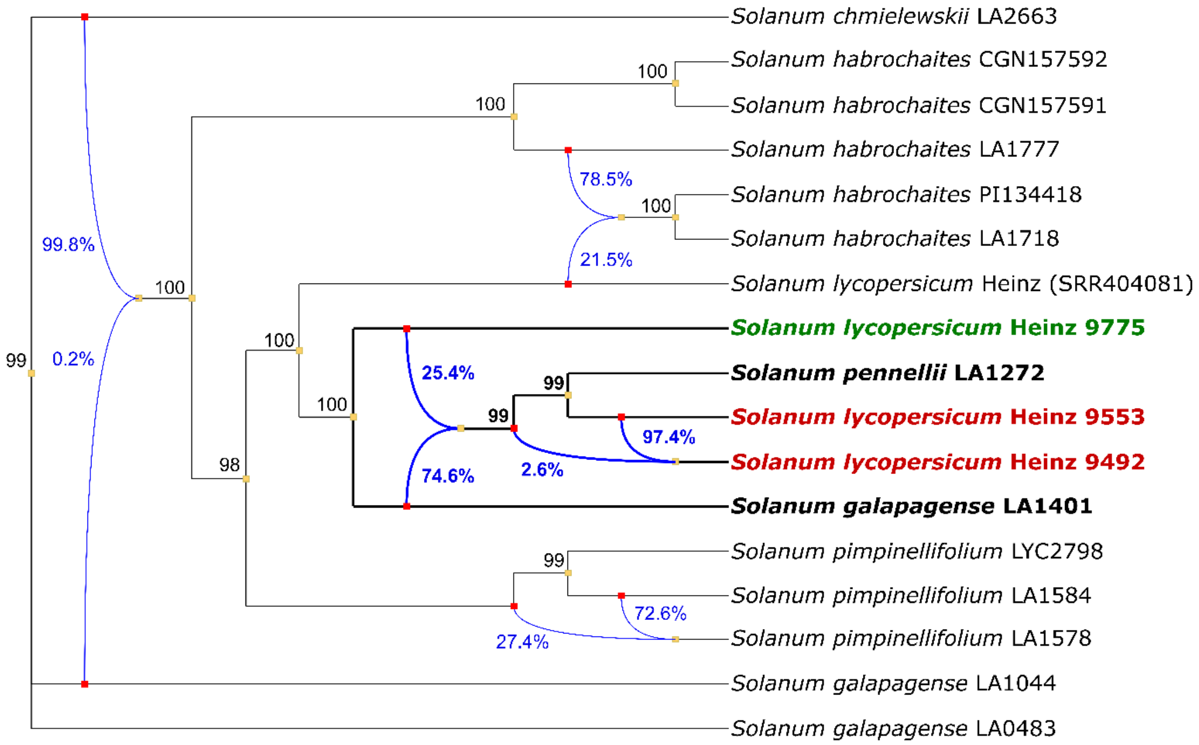

Supplemental Figure S8 | Phylogenetic network analysis using 500 kb sequence around the *LIF1* resistance-specific SNP enriched region. This phylogenetic network analysis was conducted using PhyloNetworks in the Julia environment with 500 kb of sequence around the *LIF1* resistance-specific SNP enriched region (SL3.0 ch02: 43800000 – 44300000). The species and accessions relevant to this work are highlighted in bold front and darker lines. Blue lines indicate potential hybridization events among these tomato cultivars, accessions, and species. Red nodes indicate potential hybridization parent cultivars. Blue percentage numbers represent the gene flow from each potential parent cultivar to the hybridization event. Black numbers next to each node are bootstrap values. Green bold labeled cultivar H9775 is susceptible to *C. campestris* infection. Red bold

labeled cultivars H9553 and H9492 are resistant to *C. campestris* infection. Black bold labeled species are potential tomato wild species introgression sources contributing to the *LIF1* resistance-specific SNP enriched region.

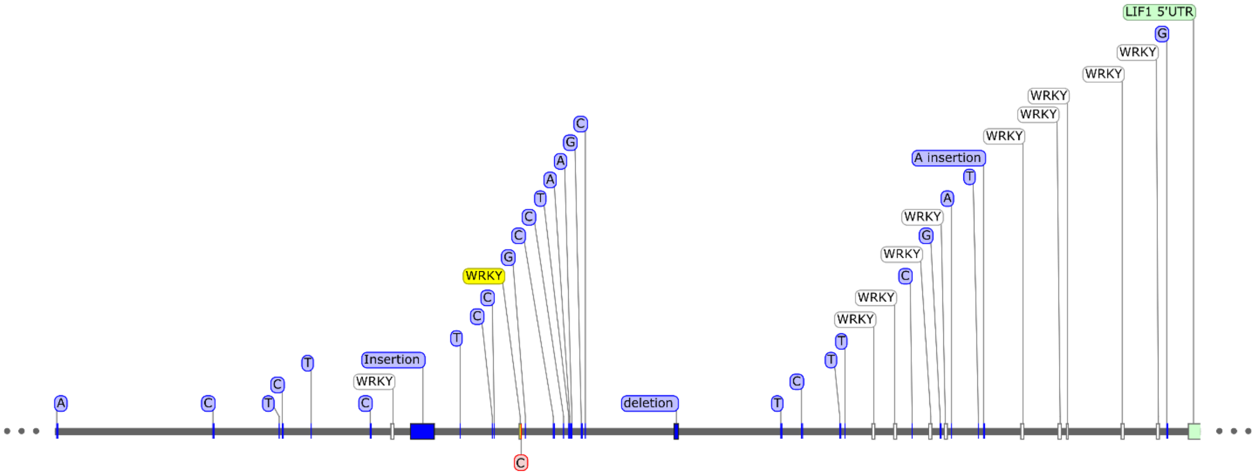

Supplemental Figure S9 | *LIF1* promoter region and transcription factors binding motifs. The *LIF1* promoter DNA sequence is labeled as a gray colored line. Transcription factors binding motifs are labeled as white boxes on the DNA sequence, and the potential key WRKY binding site is labeled as a yellow box. The 5' untranslated region (UTR) of *LIF1* gene is labeled as a green box. Resistance specific SNPs are labeled as blue lines on the DNA sequence. The resistance specific SNP that is located on the key WRKY binding site is labeled in red color.

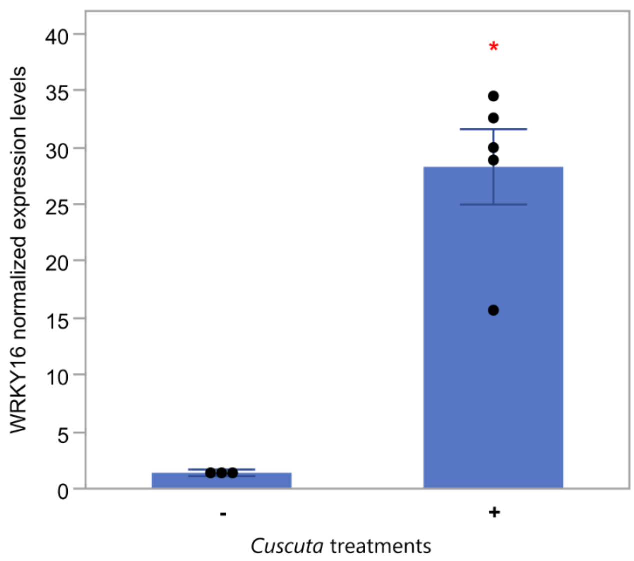

Supplemental Figure S10 | *SlWRKY16* expression levels under different *Cuscuta* treatment conditions. Normalized *SlWRKY16* expression level from RT-qPCR data in M82 tomatoes with/without *Cuscuta* treatments. – and + indicates without or with *C. campestris* infection treatments respectively. Biologically independent replicates: M82-Cc, n = 3; M82+Cc, n = 5. Data presented are assessed using two-tailed t test. "*": p-values < 0.01. Value of the t-statistic: 8.10; degrees of freedom: 4.06. The error bars indicate standard errors.

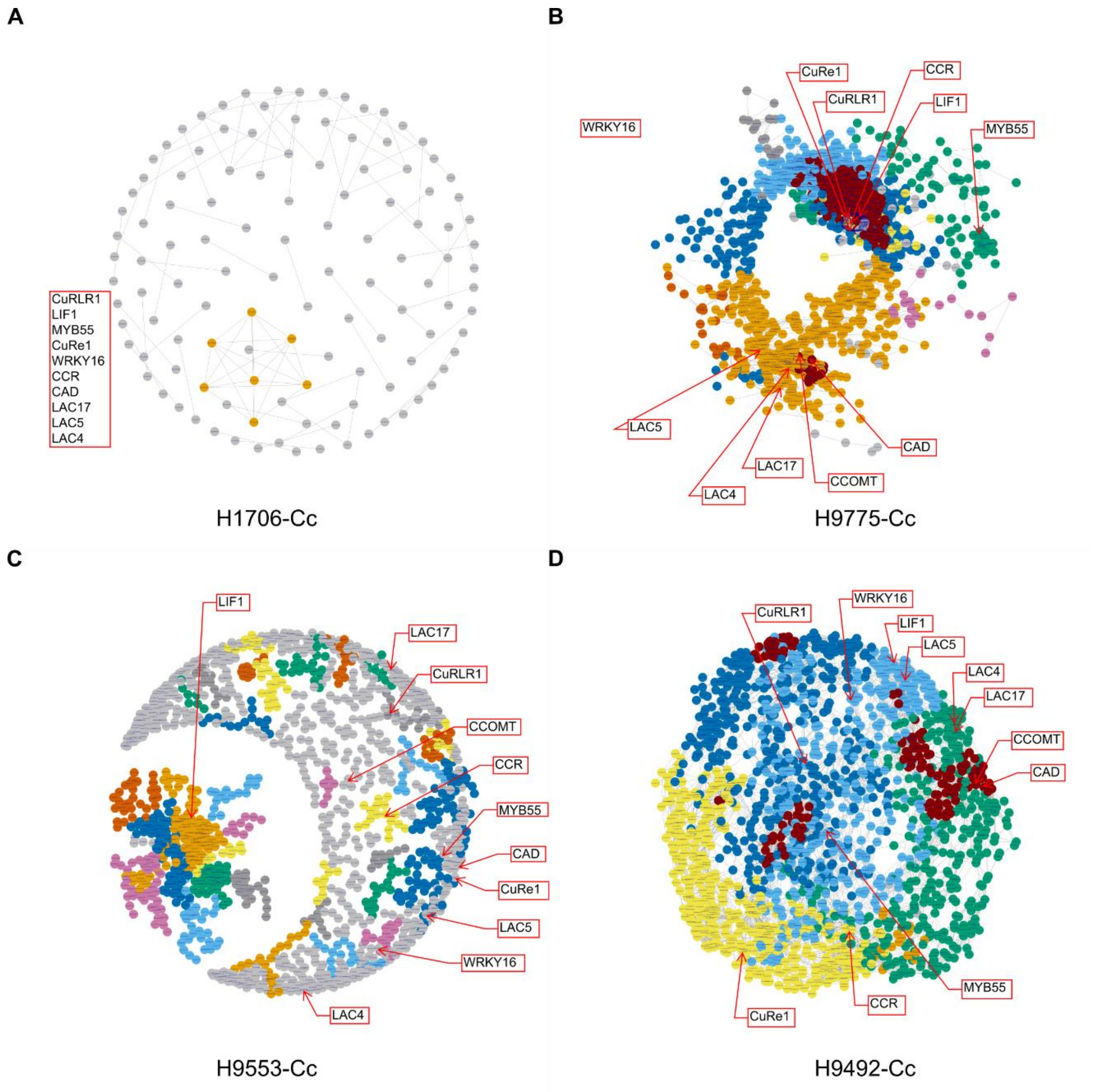

Supplemental Figure S11 | Gene co-expression network (GCN) analysis of identified key regulators. Gene co-expression networks (GCNs) of four different Heinz susceptible and resistant cultivars without *C. campestris* treatments. Based on Barnes-Hut t-distributed stochastic neighbor embedding (BH t-SNE) analysis, 1676 genes in cluster 11, 17, 23, 39, 46 and CuRLR1 are selected for building GCNs. -Cc indicate without *C. campestris* infection treatments. The genes that are listed at the left of the GCN and not labeled in the

network are the genes that have no coexpression connection with all the other genes in list. Differnt colors of the nodes indicate different modules based on GCN community structure.

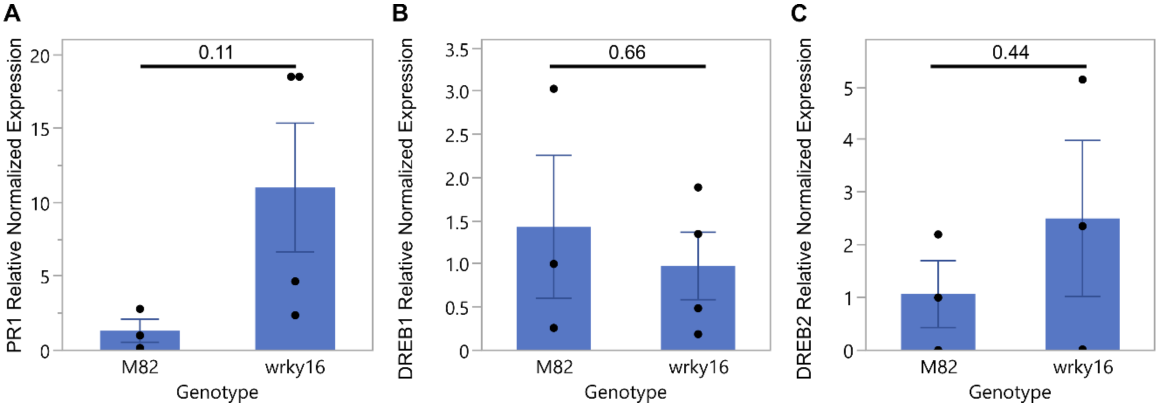

Supplemental Figure S12 | *PR1*, *DREB1*, *DREB2* expression levels in *wrky16* and M82. Normalized expression level from RT-qPCR data in M82 and *wrky16* tomatoes for (A) *PRI*. Replicates: M82, n = 3; *wrky16*, n = 4. (B) *DREB1*. Replicates: M82, n = 3; *wrky16*, n = 4. (C) *DREB2*. Replicates: M82, n = 3; *wrky16*, n = 3. Data presented are assessed using two-tailed Student's t test and p-value is labeled above the boxplot. The error bars indicate standard errors.

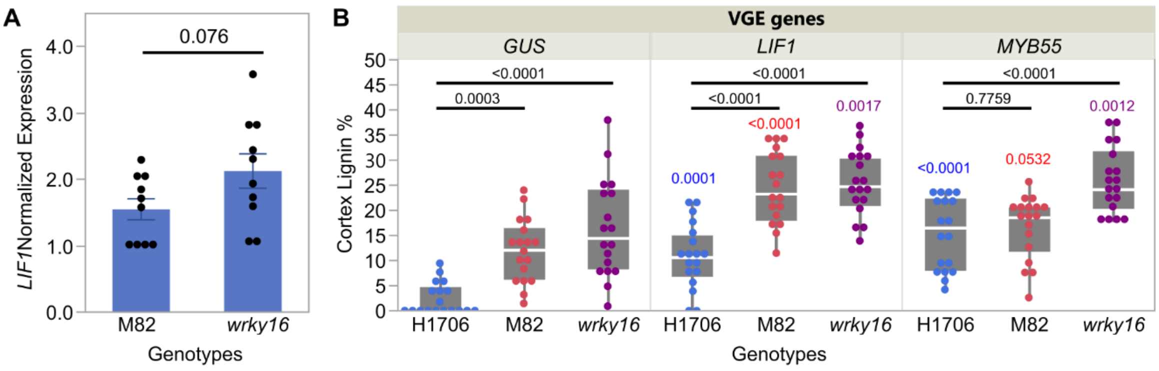

Supplemental Figure S13 | *LIF1* expression levels in *wrky16* and Virus-based Gene Expression (VGE) overexpressing *SlMYB55* and *LIF1* in H1706, M82, and *wrky16*. (A) Normalized *LIF1* expression level from RT-qPCR data in M82 and *wrky16* tomatoes. Data presented are assessed using Student's t test and p-value is labeled above the boxplot. Replicates: n = 10 for each plant genotype. (B) VGE overexpressing *SlMYB55* and *LIF1* in both susceptible H1706 and M82 tomatoes, and resistant *wrky16*. Data presented are assessed using Dunnett's test with H1706 as the control in each VGE overexpressing group and p-values are labeled above the boxplot in black. Data presented are also assessed using Dunnett's test with *GUS* as the negative control for each plant genotype. Each plant genotype group is labeled in specific color (H1706 in blue, M82 in red, *wrky16* in green) and p-values are labeled above the boxplot in the corresponding color. Replicates: n = 18 for each treatment. The boxplot consists of a box extending from the 25th quantile to the 75th quantile. The centerline in the box indicates the median. The length of the box is the interquartile range (IQR), which is the difference between the 25th quantile and the 75th quantile. The whiskers extend from the ends of the box to the outermost data point that falls within 1.5 times of IQR. Points outside of the whiskers are outliers.

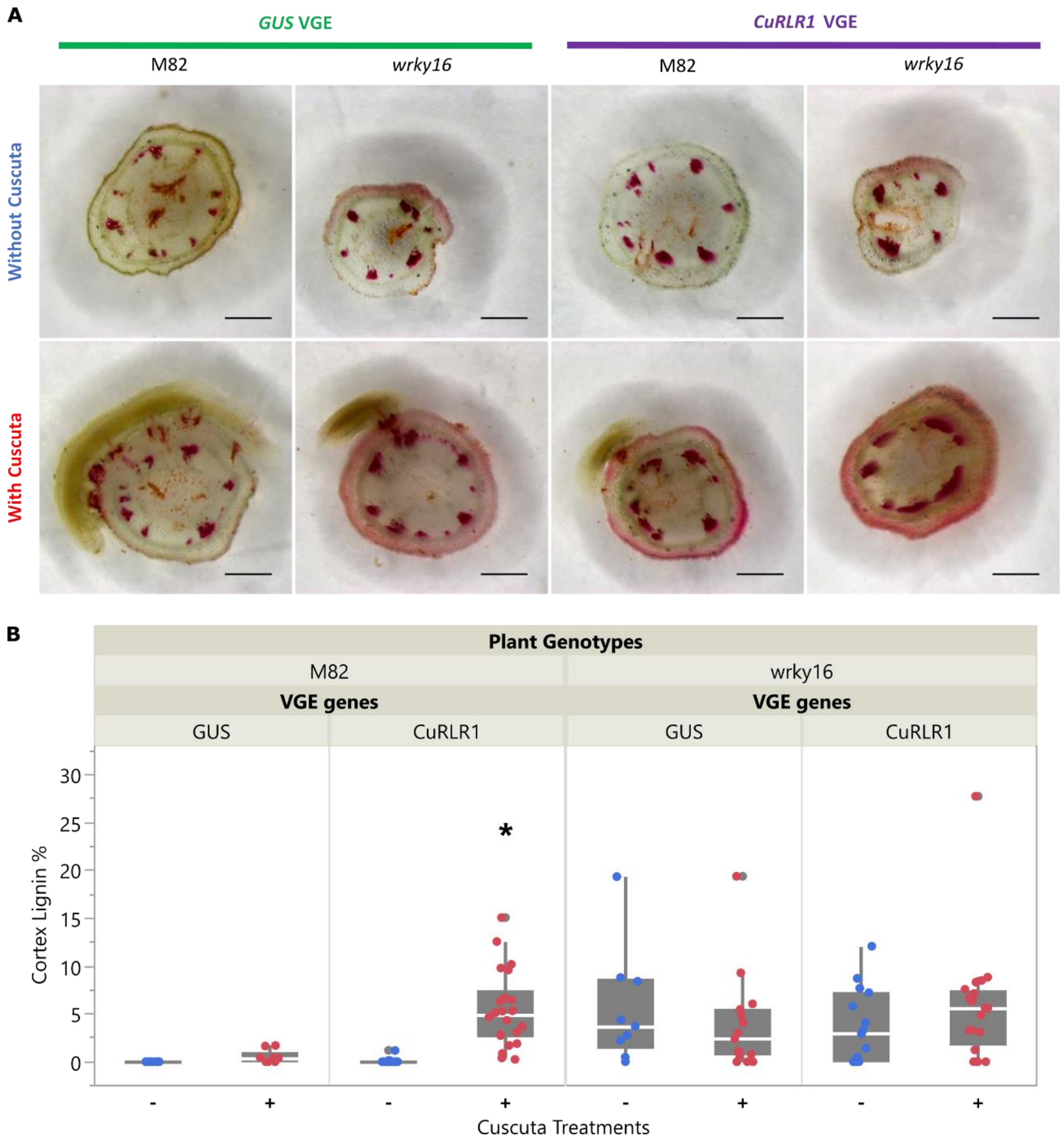

Supplemental Figure S14 | VGE overexpressing *CuRLR1* induced stem lignification in susceptible M82 tomatoes but not in *wrky16* tomatoes. (A) ~300 µm sections of the haustoria attachment sites stained with Phloroglucinol-HCl. Scale bar, 1 mm. (B) Cortex lignin area percentage in both susceptible M82 tomatoes and wrky16 tomatoes. – and + indicates without or with *C. campestris* infection treatments respectively. Data are assessed using Dunnett's test with *GUS*-Cc as the negative control for each genotype. "*": p-values

< 0.01. Replicates: M82+*GUS*-Cc, n = 10; M82+*GUS*+Cc, n = 9; M82+*CuRLR1*-Cc, n = 16; M82+*CuRLR1*+Cc, n = 22; *wrky16*+*GUS*-Cc, n = 9; *wrky16*+*GUS*+Cc, n = 15; *wrky16+CuRLR1*-Cc, n = 15; *wrky16+CuRLR1*+Cc, n = 20. Samples were collected at 10 DPI and 7 DPA. The boxplot consists of a box extending from the 25th quantile to the 75th quantile. The centerline in the box indicates the median. The length of the box is the interquartile range (IQR), which is the difference between the 25th quantile and the 75th quantile. The whiskers extend from the ends of the box to the outermost data point that falls within 1.5 times of IQR. Points outside of the whiskers are outliers.

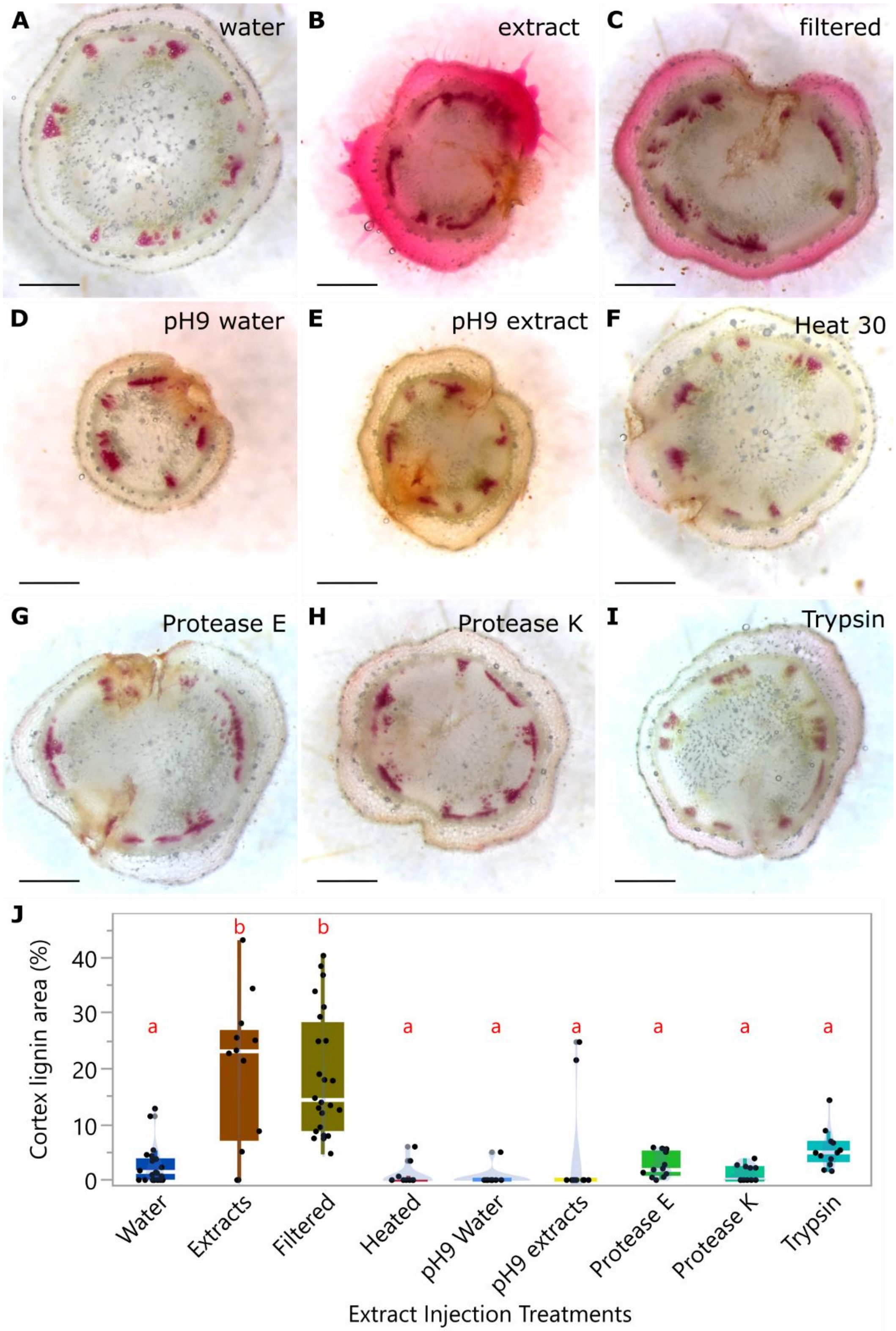
A
water
B
extract
C
filtered
D
pH9 water
E
pH9 extract
F
Heat 30
G
Protease E
H
Protease K
I
Trypsin
J
Cortex lignin area (%)
0
10
20
30
40
a
b
b
a
a
a
a
a
a
Water
Extracts
Filtered
Heated
pH9 Water
pH9 extracts
Protease E
Protease K
Trypsin
Extract Injection Treatments

Supplemental Figure S15 | *C. campestris* extract injections to detect *Cuscuta* signals. (A-I) ~300 µm hand sections of resistant H9553 stems near injection sites stained with Phloroglucinol-HCl. Lignin is stained red. Scale bar, 1 mm. Data were collected at 7 days post injection (DPI). The H9553 plants are injected with (A) water, (B) untreated *C. campestris* extract, pH 5.8, (C) *C. campestris* extract filtered with 0.2 µm filter, (D) pH 9 water, and (E) pH 9 *C. campestris* extract, (F) heat-treated *C. campestris* extract (95°C for 30 minutes), (G) Protease E-treated *C. campestris* extract, (H) Protease K-treated *C. campestris* extract, and (I) Trypsin-treated *C. campestris* extract. (J) Percentage of lignified cortex area in total stem area. The samples injected with water serve as negative controls. Different treated or untreated *C. campestris* extracts are compared to negative controls. Data presented are assessed using pair-wise comparisons with Tukey test. P-value of the contrasts between "a" and "b" are less than 0.01. Replicates: water, n = 22; untreated extract, n = 13; filtered extract, n = 24; heat-treated extract, n = 12; pH 9 water, n = 11; pH 9 extract, n = 12; Protease E-treated, n = 12; Protease K-treated, n = 12; Trypsin-treated, n = 12. The boxplot consists of a box extending from the 25th quantile to the 75th quantile. The centerline in the box indicates the median. The length of the box is the interquartile range (IQR), which is the difference between the 25th quantile and the 75th quantile. The whiskers extend from the ends of the box to the outermost data point that falls within 1.5 times of IQR. Points outside of the whiskers are outliers.

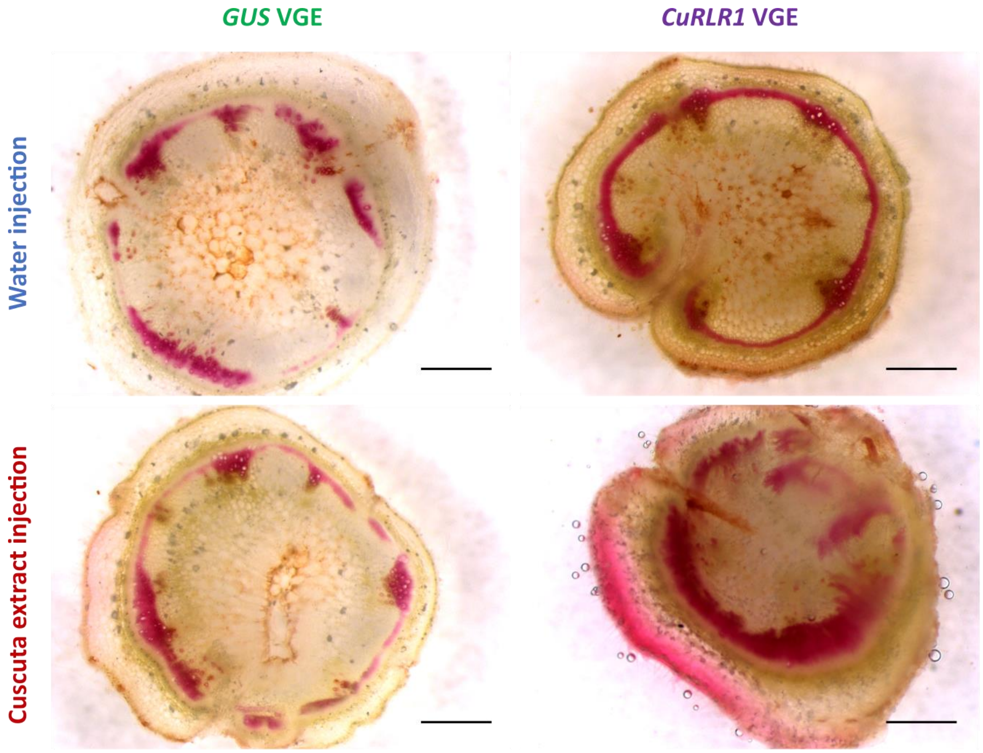

Supplemental Figure S16 | VGE overexpressing *CuRLR1* in H1706 with or without *Cuscuta* extract injections. These are ~300 µm sections of the haustoria attachment sites stained with Phloroglucinol-HCl. Scale bar, 1 mm. VGE overexpressing *GUS* served as a negative control for VGE. Water injection functions as a negative control for extract injections.

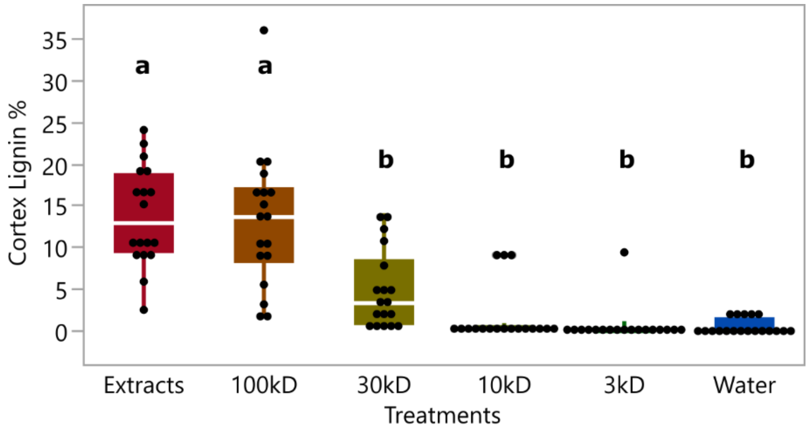

Supplemental Figure S17 | *Cuscuta* signal size analysis by using *Cuscuta* extract injections. Cortex lignin area percentage in H9553 cultivar with different size filtered Cuscuta extract injection. The *Cuscuta* extracts that flow through 3kD, 10kD, 30kD, and 100kD Amicon® Ultra Centrifugal Filter Devices are used to do injection on H9553 stems to test the size of *Cuscuta* signals. Data were assessed using pair-wise comparisons with Tukey test. Different letters indicate these groups are statistically significant different. P-values between "a" and "b" are < 0.05. Replicates: untreated extracts, n = 18; 100kD filtered extract, n = 18; 30kD filtered extract, n = 18; 10kD filtered extract, n = 18; 3kD filtered extract, n = 18; water, n = 19. The boxplot consists of a box extending from the 25th quantile to the 75th quantile. The centerline in the box indicates the median. The length of the box is the interquartile range (IQR), which is the difference between the 25th quantile and the 75th quantile. The whiskers extend from the ends of the box to the outermost data point that falls within 1.5 times of IQR. Points outside of the whiskers are outliers.

```
Cc_TrnF_Minyao       1 TTGAGCCTTGGTATGGAAACTTACTAAGTGATCACTTTCAAATTCAGAGAAACCCTGGAA
Cc_KT371721_201      1 TTGAGCCTTGGTATGGAAACTTACTAAGTGATCACTTTCAAATTCAGAGAAACCCTGGAA
Cc_KT371722_461      1 TTGAGCCTTGGTATGGAAACTTACTAAGTGATCACTTTCAAATTCAGAGAAACCCTGGAA
Cp_EF194465_456      1 TTGAGCCTTGGTATGGAAACTTACTAAGTGAGCACTTTCAAATTCAGAGAAACCCTGGAA
Cp_KT371737_1250     1 TTGAGCCTTGGTATGGAAACTTACTAAGTGAGCACTTTCAAATTCAGAGAAACCCTGGAA

Cc_TrnF_Minyao      61 TTAATAAAAAGGGCAATCCTGAGCCAAATCCTTTTTAGAATAAAAAAAAACTAAGGAAAG
Cc_KT371721_201     61 TTAATAAAAAGGGCAATCCTGAGCCAAATCCTTTTTAGAATAAAAAAAAACTAAGGAAAG
Cc_KT371722_461     61 TTAATAAAAAGGGCAATCCTGAGCCAAATCCTTTTTAGAATAAAAAAAAACTAAGGAAAG
Cp_EF194465_456     61 TTAATAAAAAAGGCAATCCTGAGCCAAATCCTTTTTAGAATAAA---AAACTAAGGAGAG
Cp_KT371737_1250    61 TTAATAAAAAAGGCAATCCTGAGCCAAATCCTTTTTAGAATAAA---AAACTAAGGAGAG

Cc_TrnF_Minyao     121 GTGCAGAGACTCAACGGAAGCTTTTCTAACCAATGTGAAAATATATTTTTTTACCAAGCT
Cc_KT371721_201    121 GTGCAGAGACTCAACGGAAGCTTTTCTAACCAATGTGAAAATATATTTTTTTACCAAGCT
Cc_KT371722_461    121 GTGCAGAGACTCAACGGAAGCTTTTCTAACCAATGTGAAAATATATTTTTTTACCAAGCT
Cp_EF194465_456    118 GTGCAGAGACTCAACGGAAGCTTTTCTAACCAATGTGAAAATATATTTTTTTACCAAGCT
Cp_KT371737_1250   118 GTGCAGAGACTCAACGGAAGCTTTTCTAACCAATGTGAAAATATATTTTTTTACCAAGCT

Cc_TrnF_Minyao     181 GATTAAAGAATAAAGAGAGAGTCCGTTTCTACATGTCAATATGGACAGTAATGAAATTTT
Cc_KT371721_201    181 GATTAAAGAATAAAGAGAGAGTCCGTTTCTACATGTCAATATGGACAGTAATGAAATTTT
Cc_KT371722_461    181 GATTAAAGAATAAAGAGAGAGTCCGTTTCTACATGTCAATATGGACAGTAATGAAATTTT
Cp_EF194465_456    178 GATTAAAGAATAAAGAGAGAGTCCGTTTCTACATGTCAATATGGACAGTAATGAAATTTT
Cp_KT371737_1250   178 GATTAAAGAATAAAGAGAGAGTCCGTTTCTACATGTCAATATGGACAGTAATGAAATTTT

Cc_TrnF_Minyao     241 CAGTAAGAAGAAAATCCGTCGATTTGAAAAATTGTGAGGGTTCAAGTCCCTCTATCCCCA
Cc_KT371721_201    241 CAGTAAGAAGAAAATCCGTCGATTTGAAAAATTGTGAGGGTTCAAGTCCCTCTATCCCCA
Cc_KT371722_461    241 CAGTAAGAAGAAAATCCGTCGATTTGAAAAATTGTGAGGGTTCAAGTCCCTCTATCCCCA
Cp_EF194465_456    238 CAGTAAGAAGAAAATCCGTCGATTTGAAAAATCGTGAGGGTTCAAGTCCCTCTATCCCCA
Cp_KT371737_1250   238 CAGTAAGAAGAAAATCCGTCGATTTGAAAAATCGTGAGGGTTCAAGTCCCTCTATCCCCA

Cc_TrnF_Minyao     301 AAAGCCCAACAAACTACTCTCTGCTTGGTACTTT-TGGCTCAACGTGCACTCCCCTTTAT
Cc_KT371721_201    301 AAAGCCCAACAAACTACTCTCTGCTTGGTACTTT-TGGCTCAACGTGCACTCCCCTTTAT
Cc_KT371722_461    301 AAAGCCCAACAAACTACTCTCTGCTTGGTACTTT-TGGCTCAACGTGCACTCCCCTTTAT
Cp_EF194465_456    298 AAAGCCCAACAAACTACTCTCT--------------------------------------
Cp_KT371737_1250   298 AAAGCCCAACAAACTACTCTCTGCTTGGTACTTTATGGCTCAAAT-TTTTTAACGTGCAC

Cc_TrnF_Minyao     360 TGGTTTCATTTCCGTTCAATCTAATCTCGAATTTGAATCTACAGTGGGAAATGGGTCGGG
Cc_KT371721_201    360 TGGTTTCATTTCCGTTCAATCTAATCTCGAATTTGAATCTACAGTGGGAAATGGGTCGGG
Cc_KT371722_461    360 TGGTTTCATTTCCGTTCAATCTAATCTCGAATTTGAATCTACAGTGGGAAATGGGTCGGG
Cp_EF194465_456    320 ---------------------TAATCTACAATTTGAATCTCCAGTGGGAAATGGGTCGGG
Cp_KT371737_1250   357 TCCTTTCATTTCCCTTCAATCTAATCTACAATTTGAATCTCCAGTGGGAAATGGGTCGGG

Cc_TrnF_Minyao     420 ATAGCTCAGGCGGTAGAGCAGAGGACTGAAAATCCTCGTGTCACCAGTTCA
Cc_KT371721_201    420 ATAGCTCAGGCGGTAGAGCAGAGGACTGAAAATCCTCGTGTCACCAGTTCA
Cc_KT371722_461    420 ATAGCTCAGGCGGTAGAGCAGAGGACTGAAAATCCT--------------C
Cp_EF194465_456    359 ATAGCTCAGTCGGTAGAGCAGAGGACTGAAAATC----------------C
Cp_KT371737_1250   417 ATAGCTCAGTCGGTAGAGCAGAGGACTGAAAATCCTCGTGT---------C
```

Supplemental Figure S18 | Sequence alignment of plastid *trnL-F* intron / spacer region sequences in our *Cuscuta campestris* isolate and published *Cuscuta campestris* and *Cuscuta pentagona*. The first sequence is from the *Cuscuta campestris* isolate we used in this research. The other sequences are from previously published *trnL-F* sequences of *Cuscuta campestris* (Cc) and *Cuscuta pentagona* (Cp) with GenBank accession numbers and following by DNA accession numbers.

```
Cc_rbcL_Minyao      1 CCGGGGCTGCAGTCGCTGCGGAATCTTCTACTGGTACATGGACAACTGTGTGGACTGATG
Cc_EU883476_411     1 CCGGGGCTGCAGTCGCTGCGGAATCTTCTACTGGTACATGGACAACTGTGTGGACTGATG
Cp_KJ436701_456     1 CCGGGGCCGCAGTCGCTGCGGAATCTTCTACTGGTACATGGACAACTGTGTGGACTGATG

Cc_rbcL_Minyao     61 GATTGACTAGCCTAGATCGGTACAAGGGTCGATGCTATCATATTGAGCGCGTGTTTGGAG
Cc_EU883476_411    61 GATTGACTAGCCTAGATCGGTACAAGGGTCGATGCTATCATATTGAGCGCGTGTTTGGAG
Cp_KJ436701_456    61 GCTTGACTAGCCTAGATCGGTACAAGGGTCGATGCTATCATATTGAGCGCGTGTTTGGAG

Cc_rbcL_Minyao    121 AAAAAGATCAATATATTGCTTATGTAGCATACCCTTTAGACCTTTTTGAAGAAGGTTCAG
Cc_EU883476_411   121 AAAAAGATCAATATATTGCTTATGTAGCATACCCTTTAGACCTTTTTGAAGAAGGTTCAG
Cp_KJ436701_456   121 AAAAAGATCAATATATTGCTTATGTAGCATACCCTTTAGACCTTTTTGAAGAAGGTTCGG

Cc_rbcL_Minyao    181 TGACCAACATGTTTACTTCAATTGTGGGGAATGTATTTGGCTTTAAAGCCCTGCGAGCTT
Cc_EU883476_411   181 TGACCAACATGTTTACTTCAATTGTGGGGAATGTATTTGGCTTTAAAGCCCTGCGAGCTT
Cp_KJ436701_456   181 TGACCAACATGTTTACTTCAATTGTGGGGAATGTTTTTGGCTTTAAAGCCCTGCGAGCTT

Cc_rbcL_Minyao    241 TACGGCTAGAAGATCTACGAATACCTCCAGCTTATACTAAAACTTTTCAAGGCCCACCTC
Cc_EU883476_411   241 TACGGCTAGAAGATCTACGAATACCTCCAGCTTATACTAAAACTTTTCAAGGCCCACCTC
Cp_KJ436701_456   241 TACGGCTCGAAGATCTACGTATACCTCCAGCTTATACTAAAACTTTTCAAGGCCCACCTC

Cc_rbcL_Minyao    301 ATGGCATCCAAGTTGAGAGAGATAAATTGAATAAATATGGCCGTCCTCTGTTGGGATGTA
Cc_EU883476_411   301 ATGGCATCCAAGTTGAGAGAGATAAATTGAATAAATATGGCCGTCCTCTGTTGGGATGTA
Cp_KJ436701_456   301 ATGGCATCCAAGTTGAGAGAGATAAATTGAATAAATATGGCCGTCCTCTGTTGGGATGTA

Cc_rbcL_Minyao    361 CTATTAAACCAAAATTGGGTTTATCAGCTAAAAATTATGGTAGAGCCGTTTATGAATGTC
Cc_EU883476_411   361 CTATTAAACCAAAATTGGGTTTATCAGCTAAAAATTATGGTAGAGCCGTTTATGAATGTC
Cp_KJ436701_456   361 CTATTAAACCAAAATTGGGTTTATCCGCTAAAAATTATGGTAGAGCCGTTTATGAATGTC

Cc_rbcL_Minyao    421 TTCGTGGTGGACTTGATTTTACCAAGGATGATGAGAATGTAAACTCACAGCCCTTTATGC
Cc_EU883476_411   421 TTCGTGGTGGACTTGATTTTACCAAGGATGATGAGAATGTAAACTCACAGCCCTTTATGC
Cp_KJ436701_456   421 TTCGTGGTGGACTTGATTTTACCAAGGATGATGAGAATGTAAACTCACAGCCCTTTATGC

Cc_rbcL_Minyao    481 GTTGGAGAGACCGTTTCCTATTTTGTGCTGAAGCAATTTATAAATCCCAAGCTGAAACCG
Cc_EU883476_411   481 GTTGGAGAGACCGTTTCCTATTTTGTGCTGAAGCAATTTATAAATCCCAAGCTGAAACCG
Cp_KJ436701_456   481 GTTGGAGAGACCGTTTCCTATTTTGTGCTGAAGCAATTTATAAATCCCAAGCTGAAACCG

Cc_rbcL_Minyao    541 GTGAAATAAAAGGACATTATTTAAATGCTACTGCAGGGACATGTGAAGAAATGCTAAGAC
Cc_EU883476_411   541 GTGAAATAAAAGGACATTATTTAAATGCTACTGCAGGGACATGTGAAGAAATGCTAAGAC
Cp_KJ436701_456   541 GTGAAATAAAAGGACATTATTTAAATGCTACTGCAGGGACATGTGAAGAAATGCTAAGAC

Cc_rbcL_Minyao    601 GAGCTTGTTTTGCTAAAGAATTGGGAGTTCCAATTATAATGCATGACTATTTAACAGGCG
Cc_EU883476_411   601 GAGCTTGTTTTGCTAAAGAATTGGGAGTTCCAATTATAATGCATGACTATTTAACAGGCG
Cp_KJ436701_456   601 GAGCTTATTTTGCTAAAGAATTGGGAGTTCCAATTATAATGCATGACTATTTAACAGGCG

Cc_rbcL_Minyao    661 GATTCACTGCAAATACTTCTTTGGCTCACTTTTGTCGAGAAAACGGGCTACTTCTTCATA
Cc_EU883476_411   661 GATTCACTGCAAATACTTCTTTGGCTCACTTTTGTCGAGAAAACGGGCTACTTCTTCATA
Cp_KJ436701_456   661 GATTCACTGCAAATACTTCTTTGGCTCACTTCTGTCGAGAAAACGGGCTACTTCTTCATA

Cc_rbcL_Minyao    721 TTCACCGTGCAATGCATGCAGTTATTGATAGACAAAAGAATCATGGGATACATTTCCGTG
Cc_EU883476_411   721 TTCACCGTGCAATGCATGCAGTTATTGATAGACAAAAGAATCATGGGATACATTTCCGTG
Cp_KJ436701_456   721 TTCACCGTGCAATGCATGCAGTTATTGATAGACAAAAGAATCATGGGATACATTTCCGCG

Cc_rbcL_Minyao    781 TACTAGCGAAGGCATTACGGTTATCTGGTGGCGATCATATTCATGCAGGTACTGTAGTAG
Cc_EU883476_411   781 TACTAGCGAAGGCATTACGGTTATCTGGTGGCGATCATATTCATGCAGGTACTGTAGTAG
Cp_KJ436701_456   781 TACTAGCGAAGGCATTACGGTTATCTGGTGGCGATCATATTCACGCAGGTACTGTAGTAG

Cc_rbcL_Minyao    841 GAAAACTGGAAGGAGAACGGGAGATTACTTTGGGCTTTGTTGACTTATTACGAGATAATT
Cc_EU883476_411   841 GAAAACTGGAAGGAGAACGGGAGATTACTTTGGGCTTTGTTGACTTATTACGAGATAATT
Cp_KJ436701_456   841 GAAAACTGGAAGGAGAACGGGAGATTACTTTGGGCTTTGTTGACTTATTACGCGATAATT

Cc_rbcL_Minyao    901 TTGTTGAAAAAGACCGAAGTCGTGGGATCTATTTTACTCAAGATTGGGTTTCGTTACCCG
Cc_EU883476_411   901 TTGTTGAAAAAGACCGAAGTCGTGGGATCTATTTTACTCAAGATTGGGTTTCGTTACCCG
Cp_KJ436701_456   901 TTGTTGAAAAAGACAGAAGTCGTGGGATCTATTTTACTCAAGATTGGGTTTCGTTACCCG

Cc_rbcL_Minyao    961 GTG
Cc_EU883476_411   961 GTG
Cp_KJ436701_456   961 GTG
```

Supplemental Figure S19 | Sequence alignment of plastid ribulose-1,5-bisphosphate carboxylase/oxygenase large subunit (*rbcL*) sequences in our *Cuscuta campestris* isolate and published *Cuscuta campestris* and *Cuscuta pentagona*. The first sequence is from the *Cuscuta campestris* isolate we used in this research. The other sequences are from previously published rbcL sequences of *Cuscuta campestris* (Cc) and *Cuscuta pentagona* (Cp) with GenBank accession numbers and following by DNA accession numbers.

```
Cc_ITS_Minyao        1 ATTATTGATTCGAATGTCTGGGTGCCGTCTTTCTGATTATGCCCACGACGAACAAAAACA
Cc_KT383150_201      1 ATTATTGATTCGAATGTCTGGGTGCCGTCTTTCTGATT-TGCCCACGACGAACAAAAACA
Cc_KT383160_461      1 ATTATTGATTCGAATGTCTGGGTGCCGTCTTTCTGATT-TGCCCACGACGAACAAAAACA
Cp_EF194664_456      1 ATTATTGATTCGAATGTCTGGGTGCCGTCTTTCTGATT-TGCCCACGACGAACAAAAACA
Cp_KT383248_1250     1 ATTATTGATTCGAGTGTCGGGGTGCTGTCTTTCTGATT-TGCCCA-GACGAACAAAAACA

Cc_ITS_Minyao       61 CCGGCGCAGCAGCGCCAAGGAATATAATAATGAGTGTGCAACCTCGCAGAGCTTGGTTAT
Cc_KT383150_201     60 CCGGCGCAGCAGCGCCAAGGAATATAATAATGAGTGTGCAACCTCGCAGAGCTTGGTTAT
Cc_KT383160_461     60 CCGGCGCAGCAGCGCCAAGGAATATAATAATGAGTGTGCAACCTCGCAGAGCTTGGTTAT
Cp_EF194664_456     60 CCGGCGCAGCAGCGCCAAGGAATATAATAATGAGTGTGCAACCTTGCAGAGCTCAATTAT
Cp_KT383248_1250    59 CCGGCGCAGCAGCGCCAAGGAATATAATAATGAGTGTGCAACCTTGCAGAGCTCAATTAT

Cc_ITS_Minyao      121 GCTGCCTGTGAGCTTTGCATCCTTTCAATAAAAATGACTCTCGGCAATGGATATCTCGGC
Cc_KT383150_201    120 GCTGCCTGTGAGCTTTGCATCCTTTCAATAAAAATGACTCTCGGCAATGGATATCTCGGC
Cc_KT383160_461    120 GCTGCCTGTGAGCTTTGCATCCTTTCAATAAAAATGACTCTCGGCAATGGATATCTCGGC
Cp_EF194664_456    120 GTTGCCTGTGAGCTTTGCATCCTTTTAATAAAAATGACTCTCGGCAATGGATATCTCGGC
Cp_KT383248_1250   119 GTTGCCTGTGAGCTTCGCATCCTTTTAATAAAAATGACTCTCGGCAATGGATATCTCGGC

Cc_ITS_Minyao      181 TCTTGCATCGATGAAGAACGTAGCGAAATGCGATACGTGGTGTGAATTGCAGAATCCCGC
Cc_KT383150_201    180 TCTTGCATCGATGAAGAACGTAGCGAAATGCGATACGTGGTGTGAATTGCAGAATCCCGC
Cc_KT383160_461    180 TCTTGCATCGATGAAGAACGTAGCGAAATGCGATACGTGGTGTGAATTGCAGAATCCCGC
Cp_EF194664_456    180 TCTTGCAACGATGAAGAACGTAGCGAAATGCGATACTTGGTGTGAATTGCAGAATCCCGT
Cp_KT383248_1250   179 TCTTGCATCGATGAAGAACGTAGCGAAATGCGATACTTGGTGTGAATTGCAGAATCCCGT

Cc_ITS_Minyao      241 GAACCATCGAAACTTTGAACGCAAGTTGCGCCTCAAGCCATTCGGTTGAGGGCACGTATG
Cc_KT383150_201    240 GAACCATCGAAACTTTGAACGCAAGTTGCGCCTCAAGCCATTCGGTTGAGGGCACGTATG
Cc_KT383160_461    240 GAACCATCGAAACTCTGAACGCAAGTTGCGCCTCAAGCCATTCGGTTGAGGGCACGTATG
Cp_EF194664_456    240 GAACCATCGAAACTTTGAACGCAAGTTGCGCCTCAAGCCATTGGGTTGAGGGCACGTCTG
Cp_KT383248_1250   239 GAACCATCGAAACTTTGAACGCAAGTTGCGCCTCAAGCCATTGGGTTGAGGGCACGTCTG

Cc_ITS_Minyao      301 CTTGGGTGTCATGCATTATGTCTCCCCTCTCGTGTGTGGAGTGGGAATAGATCCTGGCCT
Cc_KT383150_201    300 CTTGGGTGTCATGCATTATGTCTCCCCTCTCGTGTGTGGAGTGGGAATAGATCCTGGCCT
Cc_KT383160_461    300 CTTGGGTGTCATGCATTATGTCTCCCCTCTCGTGCGTGGAGTGGGAATGGATCCTGGCCT
Cp_EF194664_456    300 CTTGGGTGTCATGCACTATGTCTCCCCTCTCGTGTGTGGAGTGGGAATAGATCCTGGCCT
Cp_KT383248_1250   299 CTTGGGTGTCATGCACTATGTCTCCCCTCTCGTGTGTGGAGTGGGAATAGATCCTGGCCT

Cc_ITS_Minyao      361 CCTGGGCCCTTCCTTGGGCGTGGTTGGCCGAAAATGTTGTCCTTGATTTTGTTGATGTCT
Cc_KT383150_201    360 CCTGGGCCCTTCCTTGGGCGTGGTTGGCCGAAAATGTTGTCCTTGATTTTGTTGATGTCT
Cc_KT383160_461    360 CCTGGGCCCTTCCTTGGGCGTGGTTGGCCGAAAATGTTGTCCTTGATTTTGTTGATGTCT
Cp_EF194664_456    360 CCTGGGCCCTTCCTTGGGCGTGGTTGGCCGAAAATGTTGTCCTGGATTTTGTTGATGTCT
Cp_KT383248_1250   359 CCTGGGCCCTTCCTTGGGCGTGGTTGGCCGAAAATGTTGTCCTGGATTTTGTTGATGTCT

Cc_ITS_Minyao      421 TGGTGTGCGGTGGATGCGCCAGGTGTGCATAGTTGCCAGCCTTGCTCGGCTTCATTGTGG
Cc_KT383150_201    420 TGGTGTGCGGTGGATGCGCCAGGTGTGCATAGTTGCCAGCCTTGCTCGGCTTCATTGTGG
Cc_KT383160_461    420 TGGTGTGCGGTGGATGCGCCAGGTGTGCATAGTTGCCAGCCTTGCTCGGCTTCATTGTGG
Cp_EF194664_456    420 TGGTGTGCGGTGGATGCGCCAGGTGTGCATAGTTGCCAGCCTTGCTCGGCTTCATTGTGG
Cp_KT383248_1250   419 TGGTGAGCGGTGGATGTGCCAGGGGTGCATAGTTGCCAGCCTTGCTCGACTTCATTGTGG

Cc_ITS_Minyao      481 CGGCGGGATCCTATGAAGCTGCCGGTTTTG
Cc_KT383150_201    480 CGGCGGGATCCTATGAAGCTGCCGGTTTTG
Cc_KT383160_461    480 CGGCGGGATCCTATGAAGCTGCCGGTTTTG
Cp_EF194664_456    480 CGGCGGGATCCTATGAAGCTGCCGGTCTTG
Cp_KT383248_1250   479 CGTTGGGATCCTATGAAGCTGCCGGTTTTG
```

Supplemental Figure S20 | Sequence alignment of nuclear internal transcribed spacer (*nrITS*) sequences in our *Cuscuta campestris* isolate and published *Cuscuta campestris* and *Cuscuta pentagona*. The first sequence is from the *Cuscuta campestris* isolate we used in this research. The other sequences are from previously published *nrITS* sequences of *Cuscuta campestris* (Cc) and *Cuscuta pentagona* (Cp) with GenBank accession numbers and following by DNA accession numbers.

```
Cc_nrLSU_Minyao     1 AGCGTCCTTAGCGGCGGACTGGGCCCAAGTCCCCTGGAAAGGGGCGCCGGAGAGGGTGAG
Cc_EU883527_411     1 AGCGTCCTTAGCGGCGGACTGGGCCCAAGTCCCCTGGAAAGGGGCGCCGGAGAGGGTGAG
Cp_KJ400152_456     1 AGCGTCCTTAGCGGCGGACTGGGCCCAAGTCCCCTGGAAAGGGGCGCCGGAGAGGGTGAG

Cc_nrLSU_Minyao    61 AGCCCCGTCGGGCCTAGACCCTGTCGCACCACGAGGTGCTGTCTTAGAGTCGGGTTGTTT
Cc_EU883527_411    61 AGCCCCGTCGGGCCTAGACCCTGTCGCACCACGAGGTGCTGTCTTAGAGTCGGGTTGTTT
Cp_KJ400152_456    61 AGCCCCGTCGGGCCTAGACCCTGTCGCACCACGAGGTGCTGTCTTAGAGTCGGGTTGTTT

Cc_nrLSU_Minyao   121 GGGAATGCAGCCCAAATTTGGTGGTGAATTCCGTCCAAGGCTAAATACAGGTGAGAGCCC
Cc_EU883527_411   121 GGGAATGCAGCCCAAATTTGGTGGTGAATTCCGTCCAAGGCTAAATACAGGYGAGAGCCC
Cp_KJ400152_456   121 GGGAATGCAGCCCAAATTTGGTGGTGAATTCCGTCCAAGGCTAAATACAGGTGAGAGCCC

Cc_nrLSU_Minyao   181 GATAGCGAACAAGTACCGCGAGGGAAAGATGAAAAGAACTTTGAAAAAAGAGCCAAAGAG
Cc_EU883527_411   181 GATAGCGAACAAGTACCGCGAGGGAAAGATGAAAAGAACTTTGAAAAAAGAGCCAAAGAG
Cp_KJ400152_456   181 GATAGCGAACAAGTACCGCGAGGGAAAGATGAAAAGAACTTTGAAAAAAGAGCCAAAGAG

Cc_nrLSU_Minyao   241 TGCTTGAAATTGTCGAGAGGGAAGCGGATGGAGACCGGCGATAGGCCCAGGTTGGATGTG
Cc_EU883527_411   241 TGCTTGAAATTGTCGAGAGGGAAGCGGATGGAGACCGGCGATAGGCCCAGGTTGGATGTG
Cp_KJ400152_456   241 TGCTTGAAATTGTCGAGAGGGAAGCGGATGGAGACCGGCGATAGGCCCAGGTTGGATGTG

Cc_nrLSU_Minyao   301 GAACGGTGGTTAGCCGGTCTGCTGATAGGCTCTGGGTGTGGATCAGCGAGAATTCAGGTG
Cc_EU883527_411   301 GAACGGTGGTTAGCCGGTCTGCTGATAGGCTCTGGGTGTGGATCAGCGAGAATTCAGGTG
Cp_KJ400152_456   301 GAACGGTGGTTAGCCGGTCTGCTGATAGGCTCTGGGTGTGGATCAGCGAGAATTCAGGTG

Cc_nrLSU_Minyao   361 GCGGTTGAAGCTTGGGCATTTGATATGCTAGGGGAATGCCGTCTCTTGTATTGTGGGAAG
Cc_EU883527_411   361 GCGGTTGAAGCTTGGGCATTTGATATGCTAGGGGAATGCCGTCTCTTGTATTGTGGGAAG
Cp_KJ400152_456   361 GCGGTTGAAGCTTGGGCATTTGATATGCTAGGGGAATGCCGTCTCTTGTATTGTGGGAAG

Cc_nrLSU_Minyao   421 TAGTGCGCGCCCCGCGTGTGCTTCGGCACCTGCGTACTCAGGTCGTTGACTTGTGGGCTC
Cc_EU883527_411   421 TAGTGCGCGCCCCGCGTGTGCTTCGGCACCTGCGTACTCAGGTCGTTGACTTGTGGGCTC
Cp_KJ400152_456   421 TAGTGCGCGCCCCGCGTGTGCTTCGGCACCTGCGTACTCAGGTCGTTGACTTGTGGGCTC

Cc_nrLSU_Minyao   481 TCCATTCGACCCGTCTTGAAACACGGACCAAGGAGTCTGACATGTGTGCGAGTTGGCGAG
Cc_EU883527_411   481 TCCATTCGACCCGTCTTGAAACACGGACCAAGGAGTCTGACATGTGTGCGAGTTGGCGAG
Cp_KJ400152_456   481 TCCATTCGACCCGTCTTGAAACACGGACCAAGGAGTCTGACATGTGTGCGAGTTGGCGAG

Cc_nrLSU_Minyao   541 TGGAAAAACTCGCAAGGCGTAAGGAAGCTGATTGGCGGGATCCCTCTTTGGGGTGCACC
Cc_EU883527_411   541 TGGAAAAACTYGCAAGGCGYAAGGAAGCTGATTGGCGGGATCCCTCTTYGGGGTGCACC
Cp_KJ400152_456   541 TGGAAAAACTTGCAAGGCGCAAGGAAGCTGATTGGCGGGATCCCTCTTTGGGGTGCACC

Cc_nrLSU_Minyao   600 GTCGACCGACCTTGATCTTTTGAGAAGGGTTTGAGTGTGAGCACACCTGTTGGGACCCGA
Cc_EU883527_411   600 GYCGACCGACCTTGATCTTTTGAGAAGGGTTTGAGTGTGAGCAYACCTGTTGGGACCCGA
Cp_KJ400152_456   600 GCCGACCGACCTTGATCTTTTGAGAAGGGTTTGAGTGTGAGCATACCTGTTGGGACCCGA

Cc_nrLSU_Minyao   660 AAGATGGTGAACTATGCCTGAGCGGGGTGAAGCCAGAGGAAACTCTGGTGGAAGCCCGCA
Cc_EU883527_411   660 AAGATGGTGAACTATGCCTGAGCGGGGTGAAGCCAGAGGAAACTCTGGTGGAAGCCCGCA
Cp_KJ400152_456   660 AAGATGGTGAACTATGCCTGAGCGGGGTGAAGCCAGAGGAAACTCTGGTGGAAGCCCGCA

Cc_nrLSU_Minyao   720 GCGATACTGACGTGCAAATCGTTCGTCTGACTT
Cc_EU883527_411   720 GCGATACTGACGTGCAAATCGTTCGTCTGACTT
Cp_KJ400152_456   720 GCGATACTGACGTGCAAATCGTTCGTCTGACTT
```

Supplemental Figure S21 | Sequence alignment of nuclear large-subunit ribosomal DNA (*nrLSU*) sequences in our *Cuscuta campestris* isolate and published *Cuscuta campestris* and *Cuscuta pentagona*. The first sequence is from the *Cuscuta campestris* isolate we used in this research. The other sequences are from previously published *nrLSU* sequences of *Cuscuta campestris* (Cc) and *Cuscuta pentagona* (Cp) with GenBank accession numbers and following by DNA accession numbers.

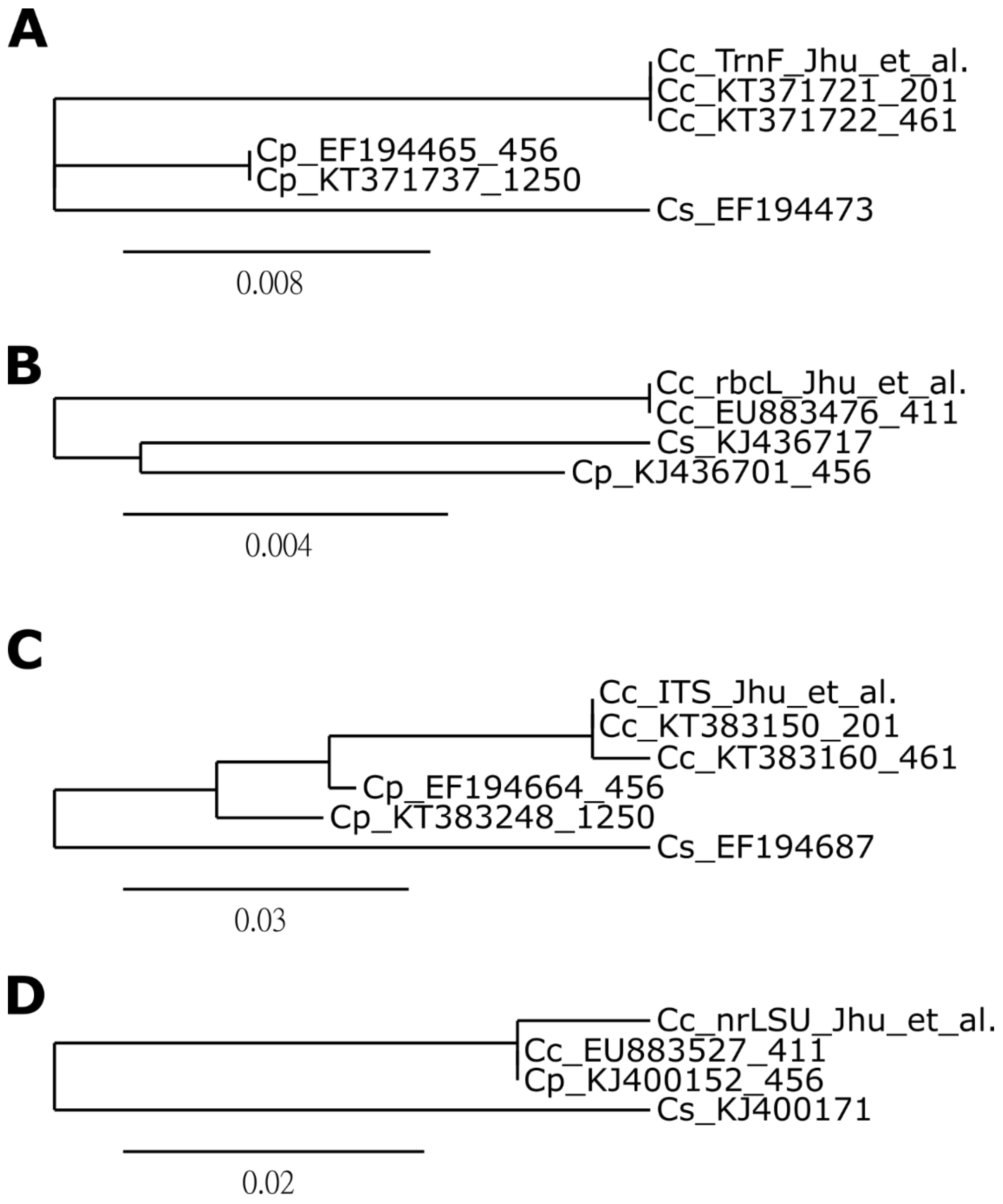

Supplemental Figure S22 | Phylogenetic relationships among our *Cuscuta campestris* isolate and published *Cuscuta campestris* and *Cuscuta pentagona* by Maximum-Likelihood Phylogenies. The first sequence of each tree is from the *Cuscuta campestris* isolate we used in this research. The other sequences are from previously published (A) *TrnL-F*, (B) *rbcL*, (C) *nrITS*, and (D) *nrLSU* sequences of *Cuscuta campestris* (Cc) and *Cuscuta pentagona* (Cp) with GenBank accession numbers and following by DNA

accession numbers. These trees are rooted using *C. stenolepis* (Cs) as functional outgroup. The number on the scale represents the percentage of genetic variation.

Supplemental Data Set S1. DEG list of time-course RNA-Seq data.

Supplemental Data Set S1 provided as an Excel file.

Supplemental Data Set S2. DEG list of resistant and susceptible host response to *C. campestris* by using an interaction design model.

Supplemental Data Set S2 provided as an Excel file.

Supplemental Data Set S3. Vector pTAV (pMR315_pTAV-GW binary) sequence

Supplemental Data Set S3 provided as a Excel file.

Supplemental Data Set S4. Haustorium infestation status quantification.

Supplemental Data Set S4 provided as an Excel file.

Supplemental Data Set S5. Resistant specific SNPs in all chromosome.

Supplemental Data Set S5 provided as an Excel file.

Supplemental Data Set S6. Resistant specific SNPs in *LIF1* promoter region.

Supplemental Data Set S6 provided as an Excel file.

Supplemental Data Set S7. Predicted transcription factor binding sites in *LIF1* promoter region.

Supplemental Data Set S7 provided as an Excel file.

Supplemental Data Set S8. DEG list of four different Heinz tomato cultivars response to *C. campestris* by ANOVA analysis.

Supplemental Data Set S8 provided as an Excel file.

Supplemental Data Set S9. Gene list in the Barnes-Hut t-distributed stochastic neighbor embedding (BH t-SNE) generated clusters.

Supplemental Data Set S9 provided as an Excel file.
